# Cryo-EM structures of biopsy-derived TTR fibrils in hereditary transthyretin amyloidosis

**DOI:** 10.1038/s41467-026-75850-8

**Published:** 2026-07-24

**Authors:** Yuxin Zheng, Jiawei Liang, Zhenyu Li, Yixiao Liu, Xujun Chu, Shaochong Zhang, Wenjuan Wang, Jionglin Bao, Boyu Liu, Conghui Ma, Guowei Yin, Jianwen Deng, Wei Chi, Lingchao Meng, Yang Shi

**Affiliations:** 1 Department of Pathology of the First Affiliated Hospital and School of Brain Science and Brain Medicine, Zhejiang University School of Medicine, Hangzhou, China; 2 Liangzhu Laboratory, Zhejiang University Medical Center, MOE Frontier Science Center for Brain Science and Brain-machine Integration, State Key Laboratory of Brain-machine Intelligence, Zhejiang University, Hangzhou, China; 3 NHC and CAMS Key Laboratory of Medical Neurobiology, Zhejiang University, Hangzhou, China; 4 Department of Neurology, Peking University First Hospital, Beijing, China; 5 Rare Diseases Medical Center, Peking University First Hospital, Beijing, China; 6 Beijing Key Laboratory of Neurovascular Disease Discovery, Beijing, China; 7 Shenzhen Eye Hospital, Shenzhen Eye Center, Southern Medical University, Shenzhen, China; 8 The First Affiliated Hospital, Guangdong Pharmaceutical University, Guangzhou, China; 9 The Seventh Affiliated Hospital of Sun Yat-sen University, Shenzhen, China; 10 School of Medicine, Sun Yat-Sen University, Shenzhen, China; 11 Present Address: Department of Neurology, Shandong Provincial Hospital Affiliated to Shandong First Medical University, Jinan, China

**Keywords:** Cryoelectron microscopy, Diagnostic markers, Peripheral neuropathies

## Abstract

Hereditary transthyretin amyloidosis (ATTRv) is a fatal autosomal dominant disease characterized by systemic deposition of transthyretin (TTR) amyloid fibrils, leading to progressive neuropathy and cardiomyopathy. More than 130 pathogenic mutations in the *TTR* gene have been identified, but their roles in TTR fibril formation and disease pathogenesis remain unclear. Here, using cryo-electron microscopy (cryo-EM), we present nineteen high-resolution TTR fibril structures (1.9-3.4 Å) from gastrocnemius muscle biopsies and vitreous humor of ten living ATTRv patients carrying nine distinct heterozygous mutations. Deep-learning-based analysis of cryo-EM densities enables semi-quantitative assessment of mutant or wild-type dominance within fibrils. These compositional profiles, combined with their structures, suggest an association between disease onset and the TTR species (wild-type or mutant) that primarily initiates amyloid formation. This biopsy-based workflow broadens access to patient tissue for amyloid structural studies, enabling systematic investigation of heterogeneous hereditary amyloidoses and the role of mutations in amyloid formation.

## Abstract (web summary)

Hereditary transthyretin amyloidosis (ATTRv) is a fatal autosomal dominant disease characterized by systemic deposition of transthyretin amyloid fibrils. Authors present nineteen high-resolution cryo-EM fibril structures from ten living ATTRv patients carrying nine distinct heterozygous mutations.

## Introduction

Transthyretin amyloidosis (ATTR) is a systemic, life-threatening disease characterized by extracellular deposition of amyloid fibrils formed by TTR. TTR transports thyroxine and retinol-binding protein, and is primarily synthesized in the liver, choroid plexus, and retinal epithelium^1^. This disease occurs in two forms: wild-type ATTR (ATTRwt), associated with age-related destabilization of TTR tetramers^2^, and hereditary ATTR (ATTRv), resulting from over 130 autosomal-dominant mutations in the *TTR* gene^3^. ATTRwt typically presents as late-onset cardiomyopathy (after age 60) with predominant myocardial deposition of TTR, and affects up to 25% of the population over the age of 80^4^. In contrast, ATTRv displays a wide range of clinical phenotypes, affecting the heart, central and peripheral nervous systems, eyes, and kidneys^5^, with 50 years often considered the threshold distinguishing early- from late-onset cases.

Advancements in cryogenic electron microscopy (cryo-EM) have enabled the determination of amyloid fibril structures from post-mortem human brains affected by neurodegenerative diseases^6–9^. The structural polymorphism of amyloid fibrils enables amyloid proteins with identical sequences to adopt different fibril folds in distinct pathological environments, providing a structural basis for disease classification^10^. Pathogenic mutations in amyloid proteins, however, introduce an additional layer of complexity to fibril folding^11–14^, complicating the information conveyed by fibril structures. ATTRv amyloidosis, with its numerous mutations, provides a valuable system for studying how single-point mutations influence fibril formation. In previous structural studies, most ATTRv fibrils from patients carrying distinct heterozygous mutations were found to adopt structures identical to that of wild-type TTR fibrils^15–22^, with distinct folds observed only in cardiac tissue from patients with the I84S mutation^23^ and in vitreous humor from patients with the V30M mutation^24^. Despite these observations, the impact of specific mutations on fibril structures and clinical manifestations remains unclear.

Here, using minimal biopsy specimens, we determined the cryo-EM structures of TTR fibrils derived from gastrocnemius muscle biopsies of ATTRv patients carrying the V30M, V30A, F33V, K35N, T59K, E61K, G83R, A97S mutations, as well as vitreous humor from an individual with the F64S mutation. Except for ATTRv-V30M and ATTRv-F64S fibrils, which have been reported from other tissue types^15,17,22,24^, fibrils of all other ATTRv mutations have not been structurally characterized. Deep-learning-based cryo-EM density analysis enables classification of each structural type into three groups: mutant-dominant, wild-type-dominant, and mixed types composed of comparable amounts of wild-type and mutant TTR. This facilitates the investigation of relationships among mutation types, fibril compositions, fibril folds, and clinical manifestations, enhancing our understanding of the molecular basis of ATTRv amyloidosis.

## Results

### ATTRv-V30M

**Fig. 1:**
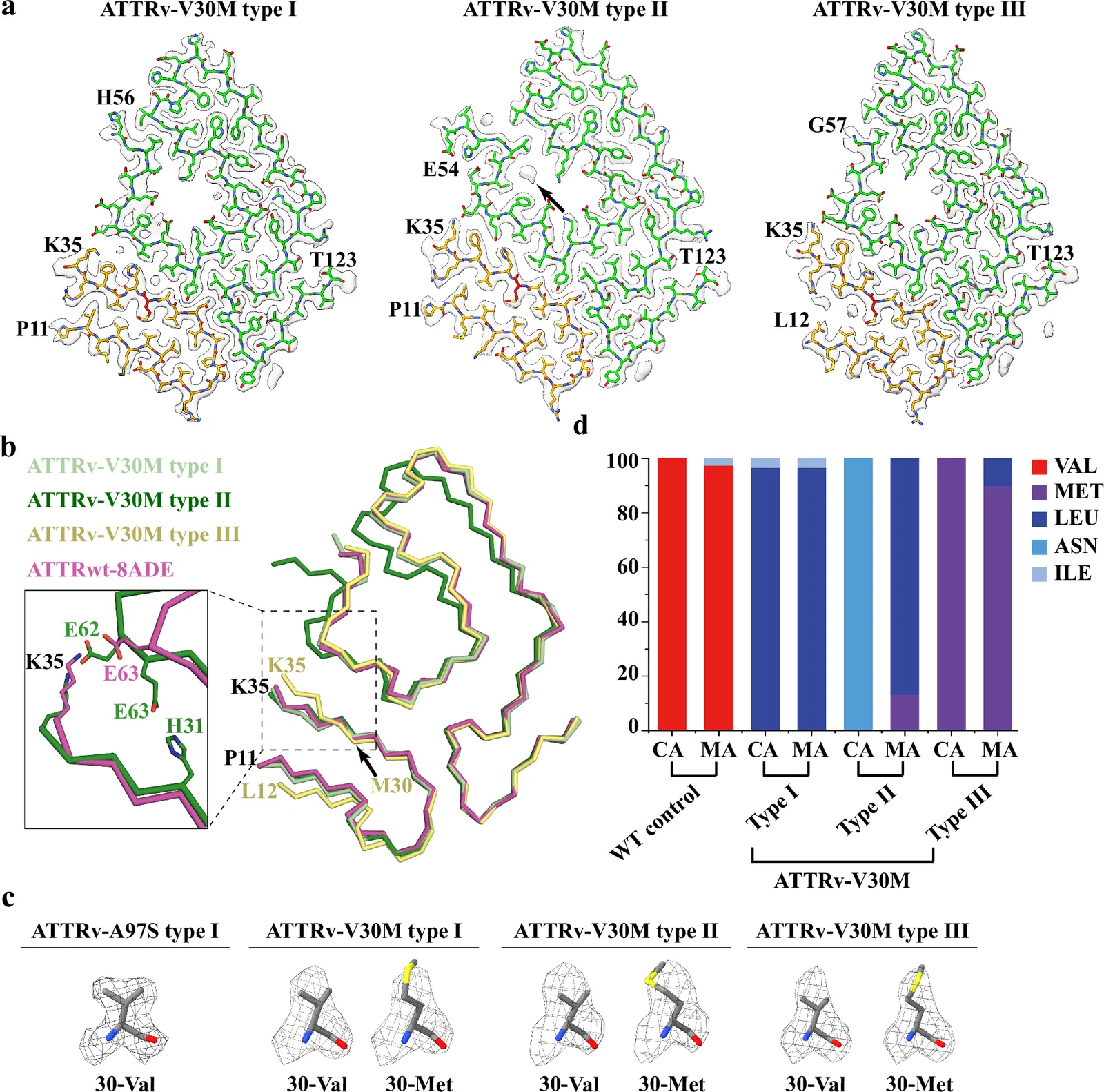
Cryo-EM structures of ATTRv-V30M fibrils. **a** Cryo-EM maps of ATTRv-V30M type I, II and III fibril structures from the gastrocnemius muscle biopsy of an individual with a V30M mutation, overlaid with corresponding atomic models. The N-terminal segment and C-terminal segment are colored orange and green, respectively. The mutation site (residue 30) is shown in red. The same color scheme is used in the following figures. Extra density is indicated by the black arrow. **b** Structural comparisons of ATTRv-V30M type I (pale green), type II (dark green), type III (yellow), and ATTRwt (8ADE, magenta) folds. The structures within the dashed boxes are realigned, on the basis of the local structural similarities. **c** Cryo-EM maps of residue 30 from ATTRv-V30M overlaid with models of valine and methionine, with the corresponding residue from the ATTRv-A97S type I fibril map included. **d** Frequencies of the most probable amino acids at residue 30 for each of the 30 rungs in control and ATTRv-V30M fibril maps, as predicted by CryoAtom (CA) and ModelAngelo (MA). Mutant amino acid (methionine) frequencies are shown in purple; wild-type (valine) frequencies in red. Raw data are provided in Supplementary Data 2.

To extract sufficient TTR fibrils from minimal biopsy tissue for cryo-EM analysis, we simplified the fibril extraction procedure^25^ by skipping multiple water extraction steps to minimize fibril loss (see Methods). Using a gastrocnemius muscle biopsy from a late-onset ATTRv-V30M patient (Supplementary Table 1 and Supplementary Fig. 1a), the most prevalent and extensively studied ATTRv mutation^15,17,24^, we successfully identified three TTR fibril structures at resolutions up to 2.49 Å, two of which have not been previously reported (Fig. 1a, Supplementary Figs. 1b, 2–4 and Supplementary Data 1).

The majority of ATTRv-V30M segments (type I) are structurally identical to the wild-type ATTR (ATTRwt) fibrils^16,18^ (hereafter, this fold is referred to as “ATTRwt fold”), with backbone root mean square deviation (RMSD) of 0.713 Å (Fig. 1a, b and Supplementary Table 2). Same to ATTRwt fold, the structure comprises two discontinuous segments: an N-terminal β‑arch spanning residues P11 to K35, and a C-terminal segment from H56 to T123. In type II structure, the inter-segment interaction is rearranged, with the K35–E63 salt bridge seen in the ATTRwt fold replaced by a K35–E62 interaction, while E63 instead forms a new interaction with H31 (Fig. 1b). The changes in the C-terminal segment of the type II structure include an extension by three residues at the N-terminus and the flipped side chains of T59, T60, and E61. Notably, an extra density was observed in the central cavity of the C-terminal segment, around residues L58, T60, and F64. Type III structure was distinguished by a widened N-terminal β‑arch and the insertion of F33 side-chain into a groove formed by the side-chain of E63 and V65 (Fig. 1a).

In all structural types, the side-chain densities at residue 30 appeared longer than those of neighboring valines (such as V28 and V32), particularly in the type III structure (Fig. 1a, c). These observations indicate the presence of V30M mutants in all ATTRv-V30M structural types. Given that cryo-EM density maps represent an average of all TTR molecules contributing to each structural type, the variation in side-chain lengths suggests differing mutant occupancies across structural types.

To objectively interpret these density variations, we used deep-learning-based structure modeling tools, CryoAtom (CA)^26^ and ModelAngelo (MA)^27^ to predict amino acid identities at the mutation sites based on the cryo-EM densities. For each structural type, the frequency of the most probable amino acid at each of the 30 rungs was used to evaluate the composition (Fig. 1d and Supplementary Data 2), with the full probability profiles provided (Supplementary Fig. 6 and Supplementary Data 3, 4).

Nine ATTR fibril maps—including the best maps from each mutation group in this study and two published maps (ATTRwt: EMD-15361; ATTRv-G47E: EMD-17737), all lacking mutations at residue 30, were used as controls (see full list in Supplementary Data 3). Valine was predicted as the most probable amino acid at residue 30 in 100% and 97% rungs of nine control maps by the MA and CA, respectively (Fig. 1d and Supplementary Data 2). In contrast, none of the layers in the three ATTRv-V30M structural types showed valine as the most probable amino acid at residue 30 (Fig. 1d and Supplementary Data 2). In type III structure, methionine—the mutant amino acid—was predicted at residue 30 in most rungs (CA: 100%; MA: 90%), suggesting the dominance of the mutant protein. In type I structure, leucine was predominantly predicted at residue 30 (CA: 97%; MA: 97%), whereas in type II structure, asparagine (CA: 100%) or leucine (MA: 87%) was assigned as the most probable residue. The side-chain sizes of leucine and asparagine are intermediate between those of valine and methionine, indicating that the observed densities at residue 30 in types I and II structures represent the averages of both wild-type and mutant residues, reflecting comparable amounts of wild-type TTR and TTR-V30M within these structures. Given that ATTRv-V30M type I (77%) and type II (16%) structures together account for 93% of all fibril segments in the tissue (Supplementary Fig. 2), the predictions indicated that both wild-type TTR and TTR-V30M contributed substantially to the amyloid deposits in this patient. These observations demonstrate that TTR fibril composition can be objectively assessed from the cryo-EM maps and classified as wild-type–dominant, mutant–dominant, or mixed. Details of the classification criteria are provided in the Methods.

### ATTRv-G83R

ATTRv-G83R is characterized by early-onset ocular involvement and slow systemic progression, and represents the most common cause of adult-onset vitreous amyloidosis^28,29^. We examined the TTR fibrils from the gastrocnemius muscle biopsies of two ATTRv-G83R patients. Both cases presented with early-onset amyloidosis involving the vitreous, followed by progressive polyneuropathy and cardiomyopathy (Supplementary Table 1 and Supplementary Fig. 1a). We observed two types of ATTRv-G83R fibril structures in case 1: a minor population (30%) consisting of single-protofilament (type I), and a predominant population (70%) with two protofilaments (type II) (Supplementary Fig. 2). In case 2, only the type II structure was observed. These findings contrast with the reported muscular TTR fibril structures from ATTRwt and ATTRv cases, in which single-protofilament fibrils are predominant (Supplementary Figs. 1b, 2).

**Fig. 2:**
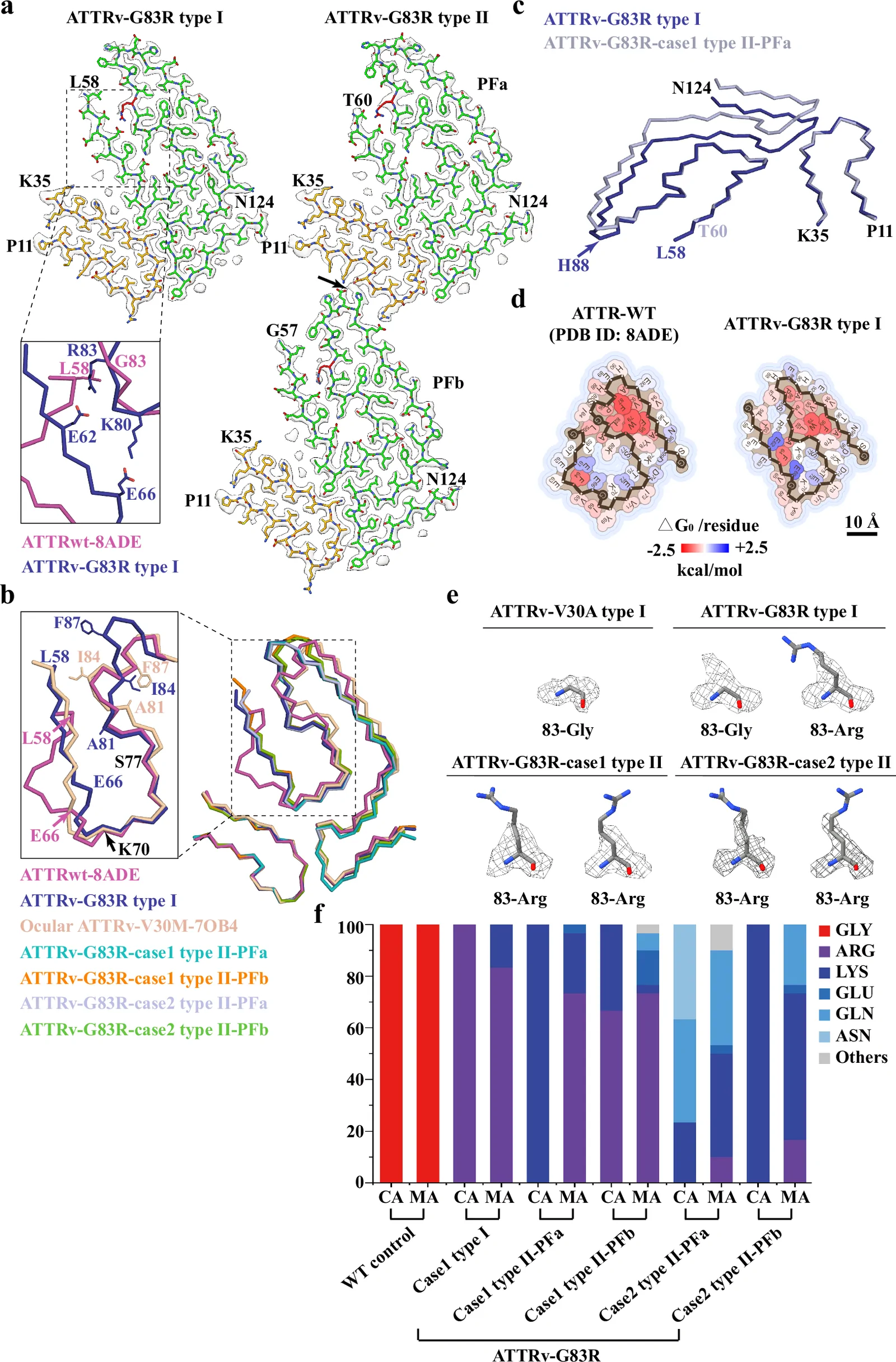
Cryo-EM structures of ATTRv-G83R fibrils. **a** Cryo-EM maps of ATTRv-G83R type I and type II fibril structures from the gastrocnemius muscle biopsy of patient 1 with the G83R mutation, overlaid with corresponding atomic models. Elongated side-chain density at H90 of ATTRv-G83R type II is indicated by a black arrow. A zoomed-in view of aligned ATTRwt and ATTRv-G83R type I structures reveals the incompatibility of the G83R mutation with the ATTRwt fold. **b** Structural comparisons of ATTRv-G83R type I (deep blue), type II from ATTRv-G83R case 1 (light teal and orange) and case 2 (light blue and green), ATTRwt (8ADE, magenta) and ocular ATTRv-V30M (7OB4, wheat) folds. The structures within the dashed box are realigned based on residues 70–77. **c** Overlay of the structures of ATTRv-G83R type Ⅰ (deep blue) and ATTRv-G83R type II-PFa (protofilament-a, slate). **d** Representation of solvation energy maps spanning residues G57 and G101 of ATTRwt (8ADE) and ATTRv-G83R type I folds. Scale bar, 10 Å. **e** Cryo-EM maps of residue 83 from ATTRv-G83R overlaid with models of glycine and arginine, with the corresponding residue from the ATTRv-V30A type I fibril map included. **f** Frequencies of the most probable amino acids at residue 83 for each of the 30 rungs in control and ATTRv-G83R fibril maps, as predicted by CryoAtom (CA) and ModelAngelo (MA). Mutant amino acid (arginine) frequencies are shown in purple; wild-type (glycine) frequencies in red. Raw data are provided in Supplementary Data 2.

In the type I structure, a long β-strand spanning residues 58–66 replaces the typical L-shaped structure seen in the ATTRwt fold, resembling the vitreous humor ATTRv-V30M fold^24^ (Fig. 2a, b and Supplementary Fig. 5). Unlike the ocular fold, however, the A81–E92 turn in the ATTRv-G83R type I structure is sharper, and the flipped I84 and F87 facilitate the formation of a β-strand from A81 to F87 (β8). Two novel salt bridges, R83-E62 and K80-E66, stabilize these structural changes (Fig. 2a). Together, these structural rearrangements accommodate the G83R substitution, which would otherwise be sterically hindered in the ATTRwt fold (Fig. 2a), suggesting that fibril formation is initiated by TTR-G83R mutants.

The type II structure from both cases adopted the same fold, with a backbone RMSD of 0.367 Å (Supplementary Table 2). The two protofilaments were arranged in a “head-to-tail” fashion, contrasting with the previously observed “head-to-head” interactions^24^. Notably, the two protofilaments in the type II structure were not identical. Protofilament a (PFa) closely resembles the ATTRv-G83R type I structure, with the only difference being the main chain shift to the adjacent rung at H88 (Fig. 2c). In contrast, protofilament b (PFb) exhibits structural changes at the inter-protofilament interface, where residues E89 and H90 shifted outward and formed interactions with K15 and D18 from PFa (Fig. 2a), likely representing an adaptive response to dimeric fibril assembly. Structural changes in both protofilaments near the mutation site suggest reduced structural stability, which is supported by the higher local solvation energy around residue 83 (residues G57–G101) in the ATTRv-G83R fold (–8.5 kcal/mol, per layer) than the corresponding region in the ATTRwt fold (–11.9 kcal/mol, per layer) (Fig. 2d).

Glycine was consistently predicted at residue 83 of all rungs in ten control maps by both CA and MA (Supplementary Data 2, 3). In contrast, in all fibrils from patient 1 and the PFb of the type II structure from patient 2, residues 83 were predominantly predicted as the mutant residue arginine, or the structurally similar lysine, with a combined prediction rate of 100% by CA and a range of 73 to 100% by MA across different structural types (Fig. 2e, f, Supplementary Fig. 6 and Supplementary Data 2, 4). These results indicate a predominance of the G83R mutant in most ATTRv-G83R fibrils. However, in PFa of the type II structure from case 2, in addition to arginine and lysine (combined prediction rates: CA, 23%; MA, 50%), glutamine (CA: 40%; MA: 37%) and asparagine (CA: 37%) were also predicted. This suggests the incorporation of wild-type TTR into these protofilaments that were initially formed by TTR-G83R mutants. Together, the fibrils in case 1 were predominantly composed of the G83R mutant TTR, whereas those in case 2 comprised a mixture of wild-type and mutant TTR.

### ATTRv-V30A and ATTRv-T59K

**Fig. 3:**
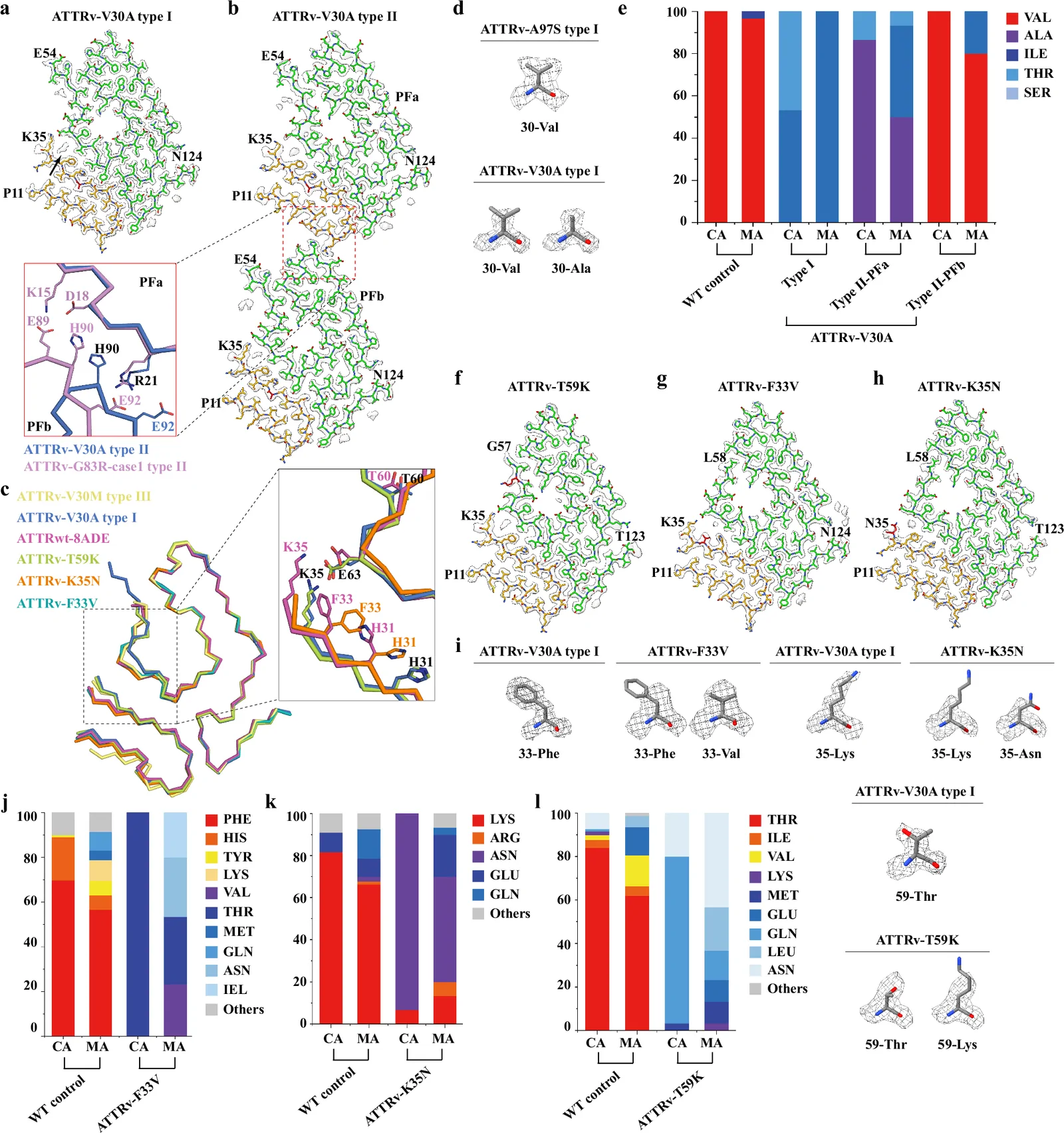
Cryo-EM structures of TTR fibrils from ATTRv patients carrying single-point mutations of V30A, F33V, K35N, and T59K. **a**, **b**, **f–h** Cryo-EM maps of ATTRv-V30A type I (**a**) and type II (**b**), ATTRv-T59K (**f**), ATTRv-F33V (**g**), ATTRv-K35N (**h**) fibril structures from the gastrocnemius muscle biopsies of patients with single-point mutations of V30A, T59K, F33V, K35N. Extra density is indicated by the black arrow in (**a**). The zoomed-in view of the interface between the protofilaments in ATTRv-V30A type II structure is shown (**b**). **c** Structural comparisons of ATTRv-V30M type III (yellow), ATTRv-V30A type I (marine), ATTRv-F33V (teal), ATTRv-K35N (orange), ATTRv-T59K (lime), and ATTRwt (8ADE, magenta) folds. The structures within the dashed boxes are realigned, on the basis of the local structural similarities. **d**, **i** Cryo-EM maps of residues at the mutation sites overlaid with models of wild-type and mutant amino acids, with the corresponding residues from ATTRv-A97S type I (**d**) and ATTRv-V30A type I (**i**) fibril maps included. **e**, **j–l** Frequencies of the most probable amino acids at residues 30 (**e**), 33 ( **j** ), 35 (**k**), 59 (**l**) for each of the 30 rungs in control and mutant TTR fibril maps, as predicted by CryoAtom (CA) and ModelAngelo (MA). In panels **j**-**l**, frequencies of the only structure identified in each case are shown: ATTRv-F33V ( **j** ), ATTRv-K35N (**k**), ATTRv-T59K (**l**). Mutant amino acid frequencies are shown in purple; wild-type frequencies in red. Raw data are provided in Supplementary Data 2.

We then examined the TTR fibrils from a patient with V30A mutation, with disease onset at age 27 (Supplementary Table 1 and Supplementary Fig. 1a). Two fibril structures were identified: the type I structure composed of a single protofilament, and the type II structure consisting of two protofilaments. Both types shared a common protofilament fold, distinct from the ATTRwt fold (Fig. 3a, b and Supplementary Figs. 1b, 2). The N-terminal β‑arch of this fold is widened, with a width intermediate between that of ATTRwt fold and ATTRv-V30M type III (Fig. 3c). The K35–E63 salt bridge was preserved, while the side chains of H31 and F33 pointed toward the closed end of the β‑arch. The change of F33 created a cavity enclosed by F33, K35, E63, and V65, within which a flat, elongated extra density was observed (Fig. 3a). The conformational changes in the C-terminal segment are characterized by the flipping of residues T59 and T60, and the formation of a new K80–E61 salt bridge. In the type II structure, two protofilaments are arranged in head-to-tail fashion, with the side chain of R21 in PFa inserting into the groove formed by the side chains of H90 and E92 in PFb, establishing a salt bridge with E92 (Fig. 3b).

In the type I structure, threonine (CA: 57%; MA: 100%) and serine (CA: 43%) were predicted in the majority rungs at residues 30 (Fig. 3d, e, Supplementary Fig. 6 and Supplementary Data 2, 4). In the type II structure, they were predicted differently in two protofilaments: in PFa, they were primarily assigned as alanine (CA: 87%; MA: 50%) or threonine (MA: 43%), while in PFb, they were predominantly predicted as valine (CA: 100%; MA: 80%). These predictions contrast with those from control maps (Supplementary Data 2, 3), where valine was consistently predicted (CA: 100%; MA: 97%), suggesting substantial contributions from both wild-type and mutant TTR to amyloid deposition in this patient.

The sub-2 Å resolution and low side-chain conformational variability of alanine and valine facilitate further quantification of the fibril composition in the ATTRv-V30A type I structure. We generated reference densities by computationally averaging cryo-EM maps of valine and alanine at a series of mixing ratios (Supplementary Fig. 7). The average Q-scores^30^ of the γ-carbon atoms (CG1 and CG2) of valine were calculated to assess the agreement between the density map and atomic model, with higher scores indicating better consistency. The Q score of a representative residue 30 in the ATTRv-V30A type I map fell between those of reference maps containing 50% and 60% alanine, suggesting a near-equal contribution of mutant (V30A) and wild-type TTR, in agreement with the residue prediction results.

Interestingly, TTR fibrils from an ATTRv-T59K patient with disease onset at age 45 (Supplementary Table 1 and Supplementary Fig. 1a) adopted the same fold as the ATTRv-V30A type I structure (Fig. 3c, f), with a backbone RMSD of 0.552 Å (Supplementary Table 2), except that the C-terminal segment was three residues shorter at the N-terminus. Residue 59 was predicted as threonine in the control maps (CA: 84%; MA: 62%, Fig. 3i, l, Supplementary Fig. 6 and Supplementary Data 2, 3), with valine also predicted by MA (14%). In contrast, the intermediate residues glutamine (CA: 77%; MA: 13%) and asparagine (CA: 20%; MA: 43%) were predicted at residues 59 in ATTRv-T59K fibrils (Fig. 3i, l, Supplementary Fig. 6 and Supplementary Data 2–4), suggesting the presence of both wild-type and mutant TTR in the fibrils of this patient.

### ATTRv-F33V and ATTRv-K35N

We then examined fibrils from two additional early-onset cases carrying the F33V and K35N mutations (Supplementary Table 1 and Supplementary Fig. 1a). In each case, only one fibril structure was observed. Both structures were highly similar to the ATTRwt fold, with backbone RMSD values of 0.800 Å and 0.774 Å, respectively (Fig. 3g, h, Supplementary Figs. 1b, 2 and Supplementary Table 2). In the ATTRv-K35N structure, the side chains of H31 and F33 were oriented toward the closed end of the β‑arch, resembling those seen in ATTRv-V30A and ATTRv-T59K structures (Fig. 3c). The densities of residue 35 in ATTRv-K35N fibrils were weaker than those in other fibril maps without the K35N mutation (Fig. 3c, h, i), consistent with the K35N mutant disrupting the salt bridge with E63, thereby increasing local flexibility.

For ATTRv-F33V fibrils, residue 33 was consistently predicted as threonine (100%) by CA, and as threonine (30%), valine (23%), and isoleucine (20%) by MA (Fig. 3i, j, Supplementary Fig. 6 and Supplementary Data 2–4). In contrast, phenylalanine was the dominant prediction in control maps (CA: 70%; MA: 57%). Given that threonine and isoleucine are structurally closer to valine than to phenylalanine, these results suggest that the fibrils were predominantly composed of the F33V mutant TTR. At residue 35, asparagine was the primary prediction for ATTRv-K35N fibrils (CA: 93%; MA: 50%), whereas lysine was largely predicted in the control maps (CA: 82%; MA: 66%; Fig. 3i, k, Supplementary Fig. 6 and Supplementary Data 2–4). As the wild-type amino acid frequency at residue 35 did not exceed 70% in MA for the control maps, ATTRv-K35N was excluded from compositional estimation. This likely reflects its position at the edge of the fibril core and its long side chain, which limit prediction accuracy.

### ATTRv-F64S

**Fig. 4:**
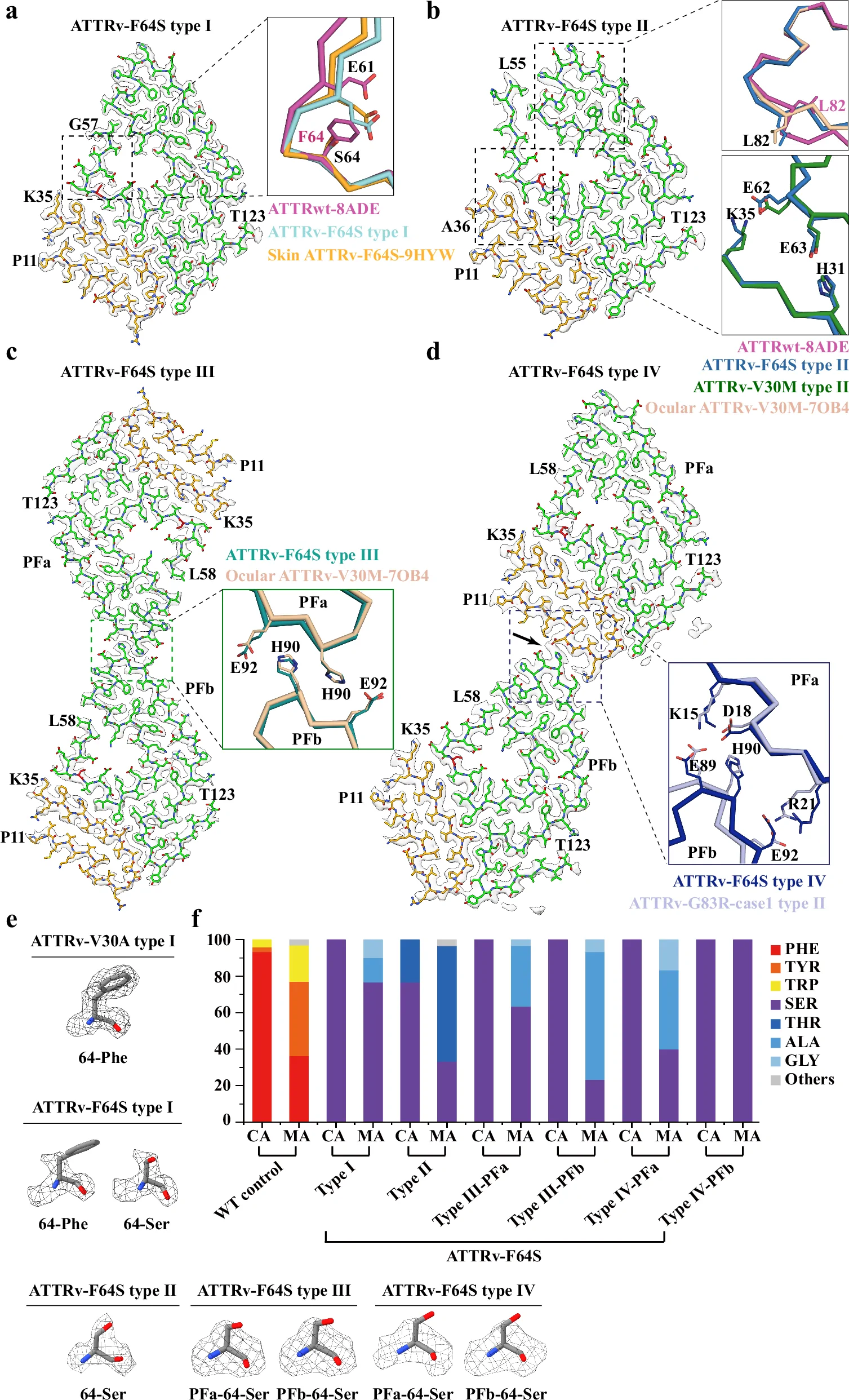
Cryo-EM structures of ATTRv-F64S fibrils. **a–d** Cryo-EM maps of ATTRv-F64S type I (**a**), type II (**b**), type III (**c**), and type IV (**d**) fibril structures from the vitreous body of an individual with an F64S mutation. The zoomed-in views of structural comparisons are also shown. Elongated side-chain density at H90 of ATTRv-F64S type IV is indicated by a black arrow (**d**). **e** Cryo-EM maps of residue 64 from ATTRv-F64S overlaid with models of phenylalanine and serine, with the corresponding residue from the ATTRv-V30A type I fibril map included. **f** Frequencies of the most probable amino acids at residue 64 for each of the 30 rungs in control and mutant TTR fibril maps, as predicted by CryoAtom (CA) and ModelAngelo (MA). Mutant amino acid (serine) frequencies are shown in purple; wild-type (phenylalanine) frequencies in red. Raw data are provided in Supplementary Data 2.

We next examined fibrils of vitreous humor from an individual with ATTRv-F64S during a vitrectomy. The patient developed progressive blurring of vision from age 27 (Supplementary Table 1 and Supplementary Fig. 1a). These fibrils comprised varying numbers of protofilaments, ranging from single to multiple protofilaments (Supplementary Fig. 1b), reminiscent of those observed in vitreous humor ATTRv-V30M fibrils^24^. We identified two single-protofilament fibril structures and two double-protofilament fibril structures (Fig. 4a–d and Supplementary Fig. 2).

The type I structure resembles the ATTRwt fold, with a backbone RMSD of 0.898 Å (Supplementary Table 2). In the N-terminal β‑arch, the β1 and β3 strands are slightly tilted toward the C-terminal segment. In the C-terminal segment, a subtle inward tilt of β4 was observed (Fig. 4a and Supplementary Fig. 5), potentially resulting from the F64S substitution, which creates additional space due to the smaller side chain of serine. Notably, this structural change would introduce steric hindrance against F64 in wild-type TTR, likely preventing its incorporation within this fold (Fig. 4a). A similar conformational feature can also be discerned in the skin-derived ATTRv-F64S structure^22^ (Fig. 4a). In the type II structure, the segmental packing resembles that of the ATTRv-V30M type II structure, with residues K35 and E62 forming a salt bridge (Fig. 4b). Residue L58, which participates in the I84-L58-A81 zipper at the “gate” of the central cavity in the ATTRwt fold, is flipped in this structure. Instead, the gate is stabilized by a steric zipper formed by residues L55, I84, and T59. Residues A81 and L82 are also flipped, resembling those in the vitreous humor ATTRv-V30M structure^24^ (Fig. 4b).

For the dimeric fibrils, we observed two structures with approximately equal proportions: the type III structure packs two protofilaments in “head-to-head” form with C2 symmetry (Fig. 4c), while the type IV structure packs two protofilaments in a “head-to-tail” form in an asymmetrical manner (Fig. 4d). All these protofilaments share the same fold as the type I structure. In the type III structure, the interactions of E92(PFa)-H90(PFb)-H90(PFa)-E92(PFb) stabilized the inter-protofilament packing (Fig. 4c), similar to that observed in the ocular ATTRv-V30M structure^24^. In the type IV structure, the protofilaments were mainly stabilized by the interaction of D18(PFa)-H90(PFb) and R21(PFa)-E92(PFb) (Fig. 4d). The twisted triple-protofilament fibrils (type V) were also observed, their high-resolution structures could not be obtained, likely due to dynamic inter-protofilament associations (Supplementary Fig. 2).

Serine is the only amino acid predicted at residue 64 in types I, III, and IV structures by CA, and is the dominant prediction in the type II structure (CA: 77%; Fig. 4e, f, Supplementary Fig. 6 and Supplementary Data 2–4). Similarly, MA predominantly predicted serine, along with alanine, threonine, and glycine, in all ATTRv-F64S structures, with combined prediction rates ranging from 97% to 100%. In contrast, phenylalanine was the primary prediction in control maps by CA, while MA assigned all aromatic residues with comparable frequencies (tyrosine: 41%, phenylalanine: 36%, tryptophan: 20%). These predictions indicated the predominance of the F64S mutant TTR in the vitreous amyloid fibrils of this patient.

In contrast to the limited muscle biopsy material, sufficient vitreous sample was available for additional bottom-up mass spectrometry (MS) analysis. Peptides containing either F64 (wild-type) or S64 (mutant) were detected, confirming the presence of both wild-type and mutant TTR (Supplementary Fig. 1c and Supplementary Table 3). The peak areas of mutant peptides were substantially higher than those of the corresponding wild-type peptides (Supplementary Fig. 1d, e). Although a single amino-acid substitution may affect ionization efficiency, this marked difference nevertheless provides supporting evidence for the dominance of mutant TTR inferred from cryo-EM maps.

### ATTRv-A97S

**Fig. 5:**
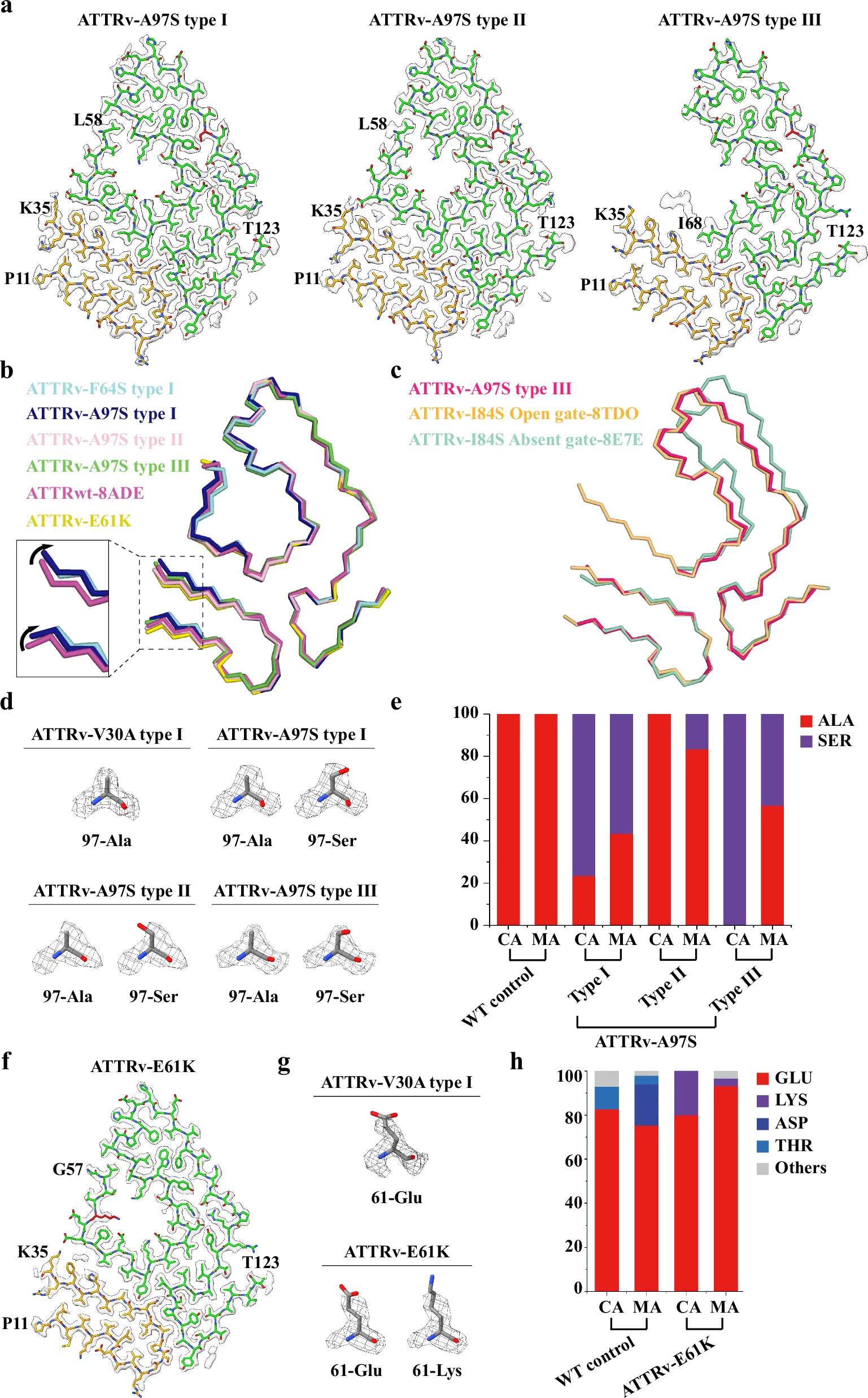
Cryo-EM structures of ATTRv-A97S and ATTRv-E61K fibrils. **a**, **f** Cryo-EM maps of ATTRv-A97S (**a**; type I, II, and III) and ATTRv-E61K (**f** ) fibril structures from gastrocnemius muscle biopsies of patients with the A97S and E61K mutations, overlaid with atomic models. **b** Structural comparisons of ATTRv-F64S type I (cyan), ATTRv-A97S type I (dark blue), ATTRv-A97S type II (light pink), ATTRv-A97S type III (green), ATTRv-E61K (yellow), and ATTRwt (8ADE, magenta) folds. **c** Structural comparisons of the ATTRv-A97S type III (hot pink) fold, ATTRv-I84S in open gate conformation (8TDO, light orange), and ATTRv-I84S in broken gate conformation (8E7E, green cyan). **d**, **g** Cryo-EM maps of residue 97 in ATTRv-A97S (**d**) and residue 61 in ATTRv-E61K (**g**) overlaid with models of wild-type and mutant amino acids, with the corresponding residues from the ATTRv-V30A type I fibril map included. **e**, **h** Frequencies of the most probable amino acids at residues 97 (**e**) and 61 (**h**) at each of the 30 rungs in control and mutant TTR fibril maps, as predicted by CryoAtom (CA) and ModelAngelo (MA). Mutant amino acid frequencies are shown in purple; wild-type frequencies in red. Raw data are provided in Supplementary Data 2.

Subsequently, we examined fibrils from the gastrocnemius muscle of a late-onset case carrying the A97S mutation, a prevalent variant in China^31^, characterized by late-onset but rapidly progressive neuropathy^32^ (Supplementary Table 1 and Supplementary Fig. 1a). Following 3D classification, we observed three single-protofilament TTR fibril structures with proportions of 85% (type I), 9% (type II), and 6% (type III) (Fig. 5a and Supplementary Fig. 2). Both type I and type II structures resemble the ATTRwt fold (RMSDs: 0.913 Å and 0.668 Å, respectively; Supplementary Table 2), with type I distinguished by a slight tilt of β1 and β3 toward the C-terminal segment, as seen in the ATTRv-F64S type I structure (Fig. 5b and Supplementary Fig. 5). In the type III structure, residues in the C-terminal segment preceding I68 are not present in the fibril core, sharing the same missing region as the broken-gate conformation of ATTRv-I84S fibrils^23^(Fig. 5c). However, residues 76–96 in the ATTRv-A97S type III structure turn inward, structurally consistent with the ATTRv-I84S open-gate conformation^23^ (Fig. 5c).

Alanine was consistently predicted at residue 97 in all control maps (CA: 100%; MA: 100%; Fig. 5d, e, Supplementary Fig. 6 and Supplementary Data 2-4). In the ATTRv-A97S type II structure, alanine remained the predominant prediction (CA: 100%; MA: 83%). In contrast, type I and III structures showed markedly increased the serine prediction rates (type I: CA, 77%; MA, 57%; type III: CA, 100%; MA, 43%), comparable to or exceeding the alanine prediction rates (type I: CA, 23%; MA, 43%; type III: CA, 0%; MA, 57%). These findings indicate that both wild-type and A97S mutant TTR contributed substantially to the amyloid deposition in this patient.

### ATTRv-E61K

Patients carrying the E61K mutation typically manifest late-onset disease, characterized primarily by sensory-motor polyneuropathy and severe cardiomyopathy^33^. In the gastrocnemius muscle of an ATTRv-E61K patient with onset at age 58 (Supplementary Table 1 and Supplementary Fig. 1a), only one fibril structure was observed, and they adopted the ATTRwt fold (Fig. 5b, f). Glutamic acid was predominantly predicted at residue 61 in both control (CA: 83%; MA: 75%) and ATTRv-E61K fibril maps (CA: 80%; MA: 93%, Fig. 5g, h, Supplementary Fig. 6 and Supplementary Data 2–4). These results suggest the dominance of wild-type TTR in these fibrils, consistent with the low amyloidogenic nature of TTR-E61K mutants^34^.

### Distinct conformations coexist within the same fibrils

For samples containing a mixture of fibril structural types, the coexistence of distinct conformers within individual fibrils has been observed in previous studies^35^. We therefore examined whether this also occurs in TTR fibrils. Following the previously reported approach^35^, we analyzed single-protofilament TTR fibrils in three cases (V30M, F64S, and A97S) exhibiting protofilament structural heterogeneity. We traced segments assigned to different structural types in the micrographs and calculated the fraction of each structural type per fibril (Supplementary Fig. 8). Although most fibrils were composed predominantly of the dominant structural type, segments corresponding to non-dominant structural types were found to coexist with other types within the same fibrils. Rather than being randomly distributed across fibrils, segments of the same structural type tended to cluster along individual fibrils. When wild-type and mutant TTR coexist, this flexible fibril organization may facilitate adaptive structural changes not only between different fibrils but also within the same fibril during elongation.

## Discussion

Structural polymorphism is a common feature for amyloid fibrils, whereby proteins with identical primary sequences can adopt distinct fibril folds. Moreover, cryo-EM structures of patient-derived fibrils are recognized as a third layer of knowledge in the characterization of proteinopathies, complementing clinical features and histopathology, and providing a structural basis for disease classification and the identification of novel disease entities^10^. In inherited proteinopathies, disease-causing mutations can further contribute to the fibril structural changes^11,12,14^. Therefore, distinguishing the underlying causes of these structural changes is critical for advancing understanding of inherited proteinopathies.

In this study, we obtained nineteen high-resolution TTR fibril structures from ten ATTRv patients carrying nine distinct mutations. Among these, fourteen exhibited structural alterations compared with the ATTRwt fold, whereas five were indistinguishable from the ATTRwt fold. Nevertheless, all retained the conserved two-segment architecture characteristic of the ATTRwt fold, consistent with previous observations^15–24^, indicating that a shared pathological mechanism underlies the formation of a common TTR fibril architecture. This contrasts with the P301L and P301T mutations in tau, which induce extensive structural rearrangements in tau fibril structures^12^. The conserved architecture of TTR fibrils may facilitate structure-guided detection of amyloidogenic conditions both in vitro and in vivo, offering key insights into the still-debated triggers of TTR aggregation^36–40^.

The mutation-induced structural changes are largely localized around the mutation sites and reflect adaptive adjustments required when the substituted amino acid is incompatible with the ATTRwt fold. Many structural alterations in our dataset are driven by steric incompatibility arising from changes in side-chain size, including increases (ATTRv-V30M type III and G83R) and decreases (ATTRv-F64S type I, III, and IV). Notably, despite a marked change in side-chain size, ATTRv-F33V fibrils adopt the ATTRwt fold, indicating that this substitution can be accommodated within the ATTRwt fold. In contrast to steric effects, changes in residue polarity and charge cause less pronounced structural changes in our dataset. No detectable structural alteration is observed in the ATTRv-E61K structure, suggesting the central cavity can accommodate the E61K substitution. Although the K35N mutation disrupts a salt bridge in the ATTRwt fold and weakens local interactions, this mutation occurs at the periphery of the fibril core and does not alter the fibril structure. G83R, involving both side-chain size and charge changes, induces the most pronounced structural alterations in our dataset. Together, these observations indicate that structural changes in TTR fibrils are determined not by the mutation itself, but by the compatibility of the substituted residue with the ATTRwt fold.

All patients in this study were heterozygous, as is typical for the vast majority of individuals with ATTRv and many other inherited proteinopathies. The co-expression of wild-type and mutant TTR further increases heterogeneity of the fibrils. Using cryo-EM map–based amino acid predictions, residues at the mutation sites were predicted as mutant, wild-type, or other amino acids with intermediate side-chain sizes. Each structural type was accordingly classified as mutant-dominant, wild-type-dominant, and hybrid. The reliability of these predictions was supported by the consistent prediction of wild-type residues at the same sites in control maps, agreement with the reference-density quantification in ATTRv-V30A type I structure, and concordance with MS data from the ATTR-F64S sample.

All wild-type-dominant structures, including ATTRv-E61K and ATTRv-A97S type II structures, adopted the ATTRwt fold (Supplementary Fig. 9). In most mutant-dominant structures—such as ATTRv-V30M type III, ATTRv-G83R (case 1), and ATTRv-F64S—structural changes are localized to the mutation sites and could be clearly attributed to steric effects introduced by the mutations, suggesting that these alterations represent localized adjustments within the ATTRwt fold to accommodate specific substitutions. Another mutant-dominant structure, ATTRv-F33V, adopted the ATTRwt fold, consistent with the high compatibility of these mutations with the ATTRwt fold. The ATTRv-F64S type II structure resembles that of the ocular ATTRv-V30M structure^24^ (Fig. 4b), which may be induced by the unique environment of the vitreous humor.

For hybrid fibrils, all structures except ATTRv-V30M type I—which adopts the ATTRwt fold—differ from the ATTRwt fold (Supplementary Fig. 9). In ATTRv-A97S type I and III, as well as ATTRv-V30M type II structures, structural changes occur far from the mutation sites, suggesting they are unlikely caused by adaptive adjustments of the ATTRwt fold to accommodate the mutations. Fibril structures from ATTRv-V30A and ATTRv-T59K, with mutations adjacent to or within regions of structural change, adopt an identical fold distinct from the ATTRwt fold, suggesting different mutations may induce the same structural changes.

In addition to protofilament structural variation, differences in the number of protofilaments and their inter-protofilament arrangements were also observed. However, the underlying mechanisms remain unclear. Non-ocular TTR fibrils are predominantly composed of single protofilaments. In contrast, ocular fibrils—from ATTRv-F64S and ATTRv-V30M cases^24^—contained a substantial proportion of dimeric and multi-protofilament fibrils, suggesting that the unique environment of the vitreous humor may favor the formation of multi-protofilament structures. In gastrocnemius muscle biopsies from ATTRv-G83R patients, dimeric fibrils represent the dominant species, potentially facilitated by mutation-induced conformational changes that destabilize the A81–E92 turn. Interestingly, additional densities next to the side chain of H90 were observed at the head-to-tail dimer interface in both ATTRv-G83R type II (Fig. 2a) and ATTRv-F64S type IV (Fig. 4d) structures, potentially corresponding to post-translational modification (PTM) moieties and suggesting the PTM-induced structural polymorphism. Similar extra densities were also observed at residue H50 in the α‑synuclein fibrils from juvenile‑onset synucleinopathy^41^.

Mass spectrometry has shown a higher proportion of mutant TTR in amyloid deposits from early-onset ATTRv-V30M cases compared to late-onset cases^42–44^. Extending this analysis to multiple mutations with cryo-EM, we estimated the patient-level TTR composition within amyloid deposits by considering both the relative abundance of each structural type and their compositions. (Supplementary Data 2, Supplementary Table 4 and Supplementary Fig. 9). In our dataset, patients whose TTR deposits are predominantly mutant tend to present with early-onset ATTRv (G83R case 1, F64S, and F33V), whereas those with predominantly wild-type deposits tend to present with late-onset cases (E61K). However, deposits containing both wild-type and mutant TTR are observed in both early- (G83R case 2, V30A and T59K) and late-onset (V30M and A97S) cases. Notably, these compositionally mixed fibrils adopted distinct folds in different patients, providing clues to the TTR species that predominates during fibril nucleation, the initial stage of fibril formation. Mixed fibrils in late-onset cases (V30M and A97S) adopt the ATTRwt fold and are likely nucleated by wild-type TTR, whereas those in early-onset cases (G83R case 2, V30A and T59K) adopt mutant-induced folds and are likely nucleated by mutant TTR. Combined analysis of fibril structures and their compositional profiles suggests that the age of disease onset correlates with the predominant TTR species driving amyloid nucleation: mutant TTR–driven amyloidosis generally presents earlier than wild-type TTR–driven disease.

In summary, by leveraging biopsy specimens and AI-assisted cryo-EM, we characterized fibril structures together with their compositional profiles, providing insights into the molecular mechanisms underlying the heterogeneity in disease onset of ATTRv amyloidosis. This approach can be broadly applied to the study and precise classification of other hereditary proteinopathies.

## Methods

### Ethics statement

The collection and use of human samples in this study were approved by the ethical review process at the Peking University First Hospital (2019-181) and the First Affiliated Hospital of Guangzhou Medical University (2024-IIT-16). Written informed consent was obtained from all patients. No residual human samples remained after the experiments.

### Source of ATTRv fibrils

Gastrocnemius muscle samples obtained during routine pathological examination from nine ATTRv patients at Peking University First Hospital (mutations: V30M, V30A, F33V, K35N, T59K, E61K, G83R [n = 2], A97S) were used for cryo-EM analysis. No additional sampling procedures were performed beyond standard clinical requirements. All these cases were reported in a multicenter study^31^. The vitreous sample of an individual with ATTRv-F64S was obtained at the First Affiliated Hospital of Guangzhou Medical University during vitrectomy. Among the ten patients, one was female and nine were male. Sex was not considered as a biological variable because of limited tissue availability. Detailed clinicopathological information is provided in Supplementary Table 1.

### Genotyping of the *TTR* gene

DNA was extracted from peripheral blood using TIANamp Genomic DNA Kit (TIANGEN, DP304), following the manufacturer’s instructions. For each patient, all four exons of the *TTR* gene were amplified by polymerase chain reaction (PCR). The PCR products were subjected to Sanger sequencing on an ABI 3730XL DNA Analyzer (Thermo Fisher Scientific). The primers (Beijing Tianyi Huiyuan Biotechnology Co., Ltd.) used and the sequencing results are provided in Source data.

### Extraction of TTR fibrils

For gastrocnemius muscle samples, TTR fibrils were extracted from fresh frozen tissue with a simplified water extraction protocol^25^. In brief, 3–40 mg of frozen muscle tissue were homogenized in Tris-calcium buffer (20 mM Tris, 138 mM NaCl, 2 mM CaCl_2_, pH 8.0), followed by centrifugation at 3100 × g for 10 min. The pellet was resuspended in Tris-calcium buffer and incubated overnight with 5 mg/mL collagenase (Sigma, 9001-12-1). After incubation, the suspension was centrifuged at 3100 × g for 30 min at 4 °C. The resulting pellet was resuspended in 20 μL of ice-cold water and used for cryo-EM analysis. Vitreous material was obtained by vitrectomy and concentrated by centrifugation at 100,000 × g for 60 min at 4 °C. The pellet was then resuspended in ice-cold water and prepared for cryo-EM.

### Mass spectrometry

A 5-μL aliquot of the fibril sample from an ATTRv-F64S patient (*n* = 1) was mixed with 65 μL water and 4 μL dithiothreitol (DTT), and then boiled at 95 °C for 5 min. Subsequently, the sample was treated with UA buffer (8 M urea, 150 mM Tris-HCl, pH 8.0) and then with SDT buffer (4% SDS, 100 mM DTT, 150 mM Tris-HCl, pH 8.0) at room temperature. The buffer exchange was performed using ultrafiltration (cut-off 10 kDa). Samples were digested overnight with trypsin (Promega) following alkylation with iodoacetamide. The resulting peptides were desalted using C18 cartridges (Sigma), then loaded onto a reverse-phase trap column (Thermo Scientific Acclaim PepMap100) and separated on a C18-reversed phase analytical column (Thermo Scientific Easy Column). Peptides were eluted using a linear gradient of buffer B (84% acetonitrile, 0.1% formic acid) in buffer A (0.1% formic acid in water) at a flow rate of 300 nL/min.

LC-MS/MS analysis was performed on a Q Exactive mass spectrometer (Thermo Scientific) operating in positive ion mode. Data were acquired using a data-dependent top 20 method which dynamically chooses the most abundant precursor ions from the survey scan (300–1800 m/z) for higher-energy collisional dissociation (HCD) fragmentation. The automatic gain control (AGC) target was set to 1 × 10^6^, with a maximum injection time of 50 ms. Peptide recognition mode was enabled during acquisition.

LC-MS/MS data were searched using MASCOT engine (Matrix Science, London, UK; version 2.2) against a non-redundant International Protein Index human sequence database (v3.85) from the European Bioinformatics Institute, supplemented with FASTA files of the mutant TTR. Database search parameters were set with a precursor tolerance of 20 ppm and a fragment ion mass tolerance of 0.1 Da. A maximum of two missed trypsin cleavages were allowed, and a minimum peptide length of 7 amino acids was used. Oxidation of methionine was included as a variable modification, and carbamidomethylation of cysteine was included as a fixed modification. Results were filtered using a false discovery rate (FDR) threshold of 1%. Ion chromatogram extraction and peak area quantification were performed using Skyline 26.1.

### Cryo-EM

Holey carbon grids (Nanodim Au R1.2/1.3, 300 mesh) were glow-discharged for 60 s using Coolglow glow discharge system operated at 40 mA. Samples were applied to glow discharged grids and plunge-frozen in liquid ethane using Thermo Scientific Vitrobot Mark IV System. Images were acquired using a 300 kV Titan Krios microscope (Thermo Fisher) equipped with a Falcon 4i. Aberration-free image shift in the EPU software (Thermo Fisher) was used during image acquisition. Further details are provided in Supplementary Data 1.

### Helical reconstruction

Datasets were processed in RELION-5.0 using standard amyloid helical reconstruction procedures^45^. Movie frames were gain corrected, aligned, and dose weighted using RELION’s own motion correction program^46^. The contrast transfer function (CTF) parameters were estimated using CTFFIND-4.1^47^. Fibrils were picked manually and extracted with a box size of 900 pixels, subsequently down-scaled to 300 pixels. Reference-free 2D classification was performed to remove low-quality segments, and representative 2D class averages for each structural type were selected to generate initial models using *relion_helix_inimodel2d*. For high-resolution refinement, selected segments from each structural type were extracted with a box size of 256 or 360 pixels at the original pixel size. 3D auto-refinements were performed, with optimization of the helical twist and rise parameters once resolutions extended beyond 4.8 Å. Bayesian polishing and CTF refinement were applied to further improve the resolution^46^. The structure heterogeneity was examined by 3D classification without alignment. Final maps were sharpened using standard postprocessing procedures in RELION, and resolution estimates were calculated based on the Fourier shell correlation (FSC) between two independently refined half-maps at 0.143^48^ (Supplementary Fig. 4). Further details are provided in Supplementary Data 1.

### Atomic modeling

Atomic models were built manually in Coot^49^ (version 0.9.8) using the published structure (PDB: 8ADE) as an initial model. Each model was then subjected to iterative cycles of automatic real-space refinements in PHENIX^50^ (version 1.21-5207-000) and manual adjustment in Coot. The mutated residues were used in the model building and the refinement. Models were validated with MolProbity^51^. The refined structures were then refined against their first half-maps in PHENIX, and the resulting models were compared to their second half-map to confirm the absence of overfitting. Further details are provided in Supplementary Data 1. Figures of structures were prepared using ChimeraX^52^ (version 1.7.1). The solvation energy maps in Fig. 2d were prepared using the Amyloid Illustrator software^53^(https://srv.mbi.ucla.edu/AmyloidAtlas/Illustrator).

### Cryo-EM density-based prediction of protein variants

The optimized helical rise and twist were applied to the post-processed cryo-EM maps using *relion_helix_toolbox*, prior to amino acid predictions with CryoAtom^26^ 1.0.0^54^ and ModelAngelo^27^ 1.0.10. The predictions were performed without incorporating sequence information. The frequencies of the most probable amino acids at each of the central 30 rungs were used for the fibril composition evaluation. The probabilities of all 20 amino acids at the mutation sites within the central 30 rungs were averaged and provided in Supplementary Data 3 and 4. For each mutation site, cryo-EM maps of TTR fibrils, with resolutions better than 3.0 Å, lacking mutations at the corresponding positions, and from different individuals were selected as the negative controls. The complete lists of these maps are provided in Supplementary Data 3.

For classification, structures in which ≥70% of residues at the mutation sites within the central 30 rungs were predicted as wild-type or mutant amino acids by both CA and MA were classified accordingly as wild-type–dominant or mutant–dominant; all other structures were classified as mixed. As local resolution varies in cryo-EM and affects prediction accuracy, sites with wild-type frequencies <70% in control maps from either CA or MA were excluded from the assessment. In some cases, amino acids with similar side chains to the target residue were grouped together during the calculation of prediction frequencies. Details are provided in Supplementary Table 4. At the patient level, compositional classification was determined by the relative proportions of segments in each category (wild-type–dominant, mutant–dominant, or mixed).

In accordance with established consensus^55,56^, a clinical threshold of 50 years was used to distinguish early-onset (<50 years) from late-onset (≥50 years) cases.

### Reference-density-based protein variants assessment

In the ATTRv-V30A type I reconstruction, residues V32 and A97 from the same map were separated, aligned, and computationally mixed at varying ratios in UCSF Chimera^57^ (version 1.17.1) to generate reference standards. The average Q-scores of the gamma carbon atoms of valine for each map, calculated using MapQ in UCSF Chimera, were used as the readout for quantification.

### Reporting summary

Further information on research design is available in the Nature Portfolio Reporting Summary linked to this article.

## Supplementary information

Supplementary Information

Description of Additional Supplementary Files

Supplementary Data 1

Supplementary Data 2

Supplementary Data 3

Supplementary Data 4

Reporting Summary

Transparent Peer Review file

## Source data

Source Data

## Data Availability

Cryo-EM maps have been deposited in the Electron Microscopy Data Bank (EMDB) under accession numbers EMD-65779 for ATTRv-V30M Type I, EMD-65780 for ATTRv-V30M Type II, EMD-65781 for ATTRv-V30M Type III, EMD-65794 for ATTRv-V30A Type I, EMD-65795 for ATTRv-V30A Type II, EMD-65797 for ATTRv-F33V, EMD-65796 for ATTRv-K35N, EMD-65790 for ATTRv-T59K, EMD-65789 for ATTRv-E61K, EMD-65782 for ATTRv-F64S Type I, EMD-65783 for ATTRv-F64S Type II, EMD-65784 for ATTRv-F64S Type III, EMD-65785 for ATTRv-F64S Type IV, EMD-65792 for ATTRv-G83R case1 Type I, EMD-65793 for ATTRv-G83R case1 Type II, EMD-65791 for ATTRv-G83R case2 Type II, EMD-65786 for ATTRv-A97S Type I, EMD-65787 for ATTRv-A97S Type II, EMD-65788 for ATTRv-A97S Type III. Corresponding refined atomic models have been deposited in the Protein Data Bank (PDB) under accession numbers 9W9J for ATTRv-V30M Type I, 9W9K for ATTRv-V30M Type II, 9W9L for ATTRv-V30M Type III, 9W9Z for ATTRv-V30A Type I, 9WA0 for ATTRv-V30A Type II, 9WA2 for ATTRv-F33V, 9WA1 for ATTRv-K35N, 9W9V for ATTRv-T59K, 9W9U for ATTRv-E61K, 9W9N for ATTRv-F64S Type I, 9W9O for ATTRv-F64S Type II, 9W9P for ATTRv-F64S Type III, 9W9Q for ATTRv-F64S Type IV, 9W9X for ATTRv-G83R case1 Type I, 9W9Y for ATTRv-G83R case1 Type II, 9W9W for ATTRv-G83R case2 Type II, 9W9R for ATTRv-A97S Type I, 9W9S for ATTRv-A97S Type II, 9W9T for ATTRv-A97S Type III. Mass spectrometry data have been deposited to the ProteomeXchange Consortium via the iProX partner repository under accession code PXD070068. The genotyping sequencing data of mutations in TTR patients have been deposited in the Genome Sequence Archive^58^ in National Genomics Data Center^59^, China National Center for Bioinformation / Beijing Institute of Genomics, Chinese Academy of Sciences (GSA-Human: HRA019078). Unless otherwise stated, all data supporting the results of this study can be found in the article, supplementary, and source data files. Source data are provided with this paper.

## Code availability

The software packages used in this work were RELION 5.0 (https://github.com/3dem/relion), CTFFIND 4.1 (https://grigoriefflab.umassmed.edu/ctf_estimation_ctffind_ctftilt), Coot 0.9.8 (https://www2.mrc-lmb.cam.ac.uk/personal/pemsley/coot/source/releases), PHENIX 1.21-5207-000 (https://phenix-online.org/download), PyMOL 3.1.1 (https://www.pymol.org/), ChimeraX 1.7.1 (https://www.cgl.ucsf.edu/chimerax/download.html), CryoAtom 1.0.0 (https://zenodo.org/records/20825279), and ModelAngelo 1.0.10 (source code: https://github.com/3dem/model-angelo; pretrained weights: https://zenodo.org/records/7942241).
