# Supplementary material for: Cryo-EM structures of biopsy-derived TTR fibrils in hereditary transthyretin amyloidosis
Source: Nat Commun. 2026 Jul 24;17:9023. doi: 10.1038/s41467-026-75850-8 (PMC13507274; doi:10.1038/s41467-026-75850-8)
Supplement: Supplementary file 1 [file 41467_2026_75850_MOESM1_ESM.pdf]

Supplementary information

Cryo-EM Structures of Biopsy-derived TTR Fibrils in Hereditary Transthyretin Amyloidosis

Table of contents:

Supplementary Figures 1-9

Supplementary Tables 1-4

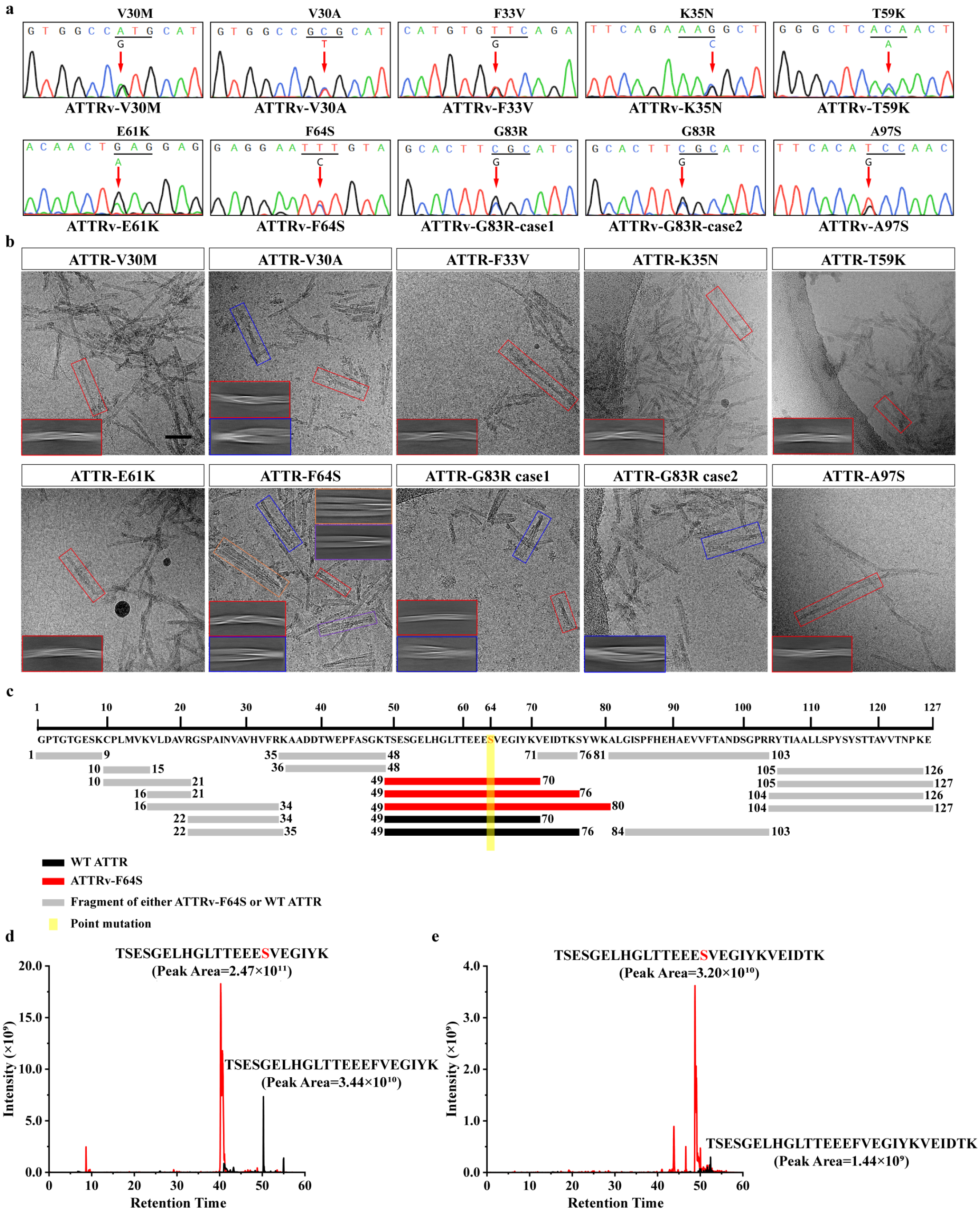

Supplementary Fig. 1 | Filamentous TTR pathology from ATTRv patients.

a, Sanger sequencing verification of ATTRv mutations in all patients. **b,** Representative cryo-EM micrographs of TTR fibrils. 2D class averages of TTR fibrils are shown at the bottom left of each panel; for ATTRv-F64S, they are also shown at the top right. Red boxes indicate fibrils with a single protofilament; blue boxes, asymmetric dimeric fibrils; purple box, symmetric dimeric fibril; orange box, trimeric fibril. Scale bar, 50 nm. For

each case, cryo-EM data were collected and processed from a single preparation. **c**, Trypsin-digested TTR fragments detected by mass spectrometry from the vitreous body of a patient carrying an F64S mutation. **d**, **e**, Extracted liquid chromatography - mass spectrometry (LC-MS) ion chromatogram of the wild-type (black) and F64S-mutant (red) peptides in fibrils from the vitreous body of a patient carrying an F64S mutation. Source data are provided as a Source Data file.

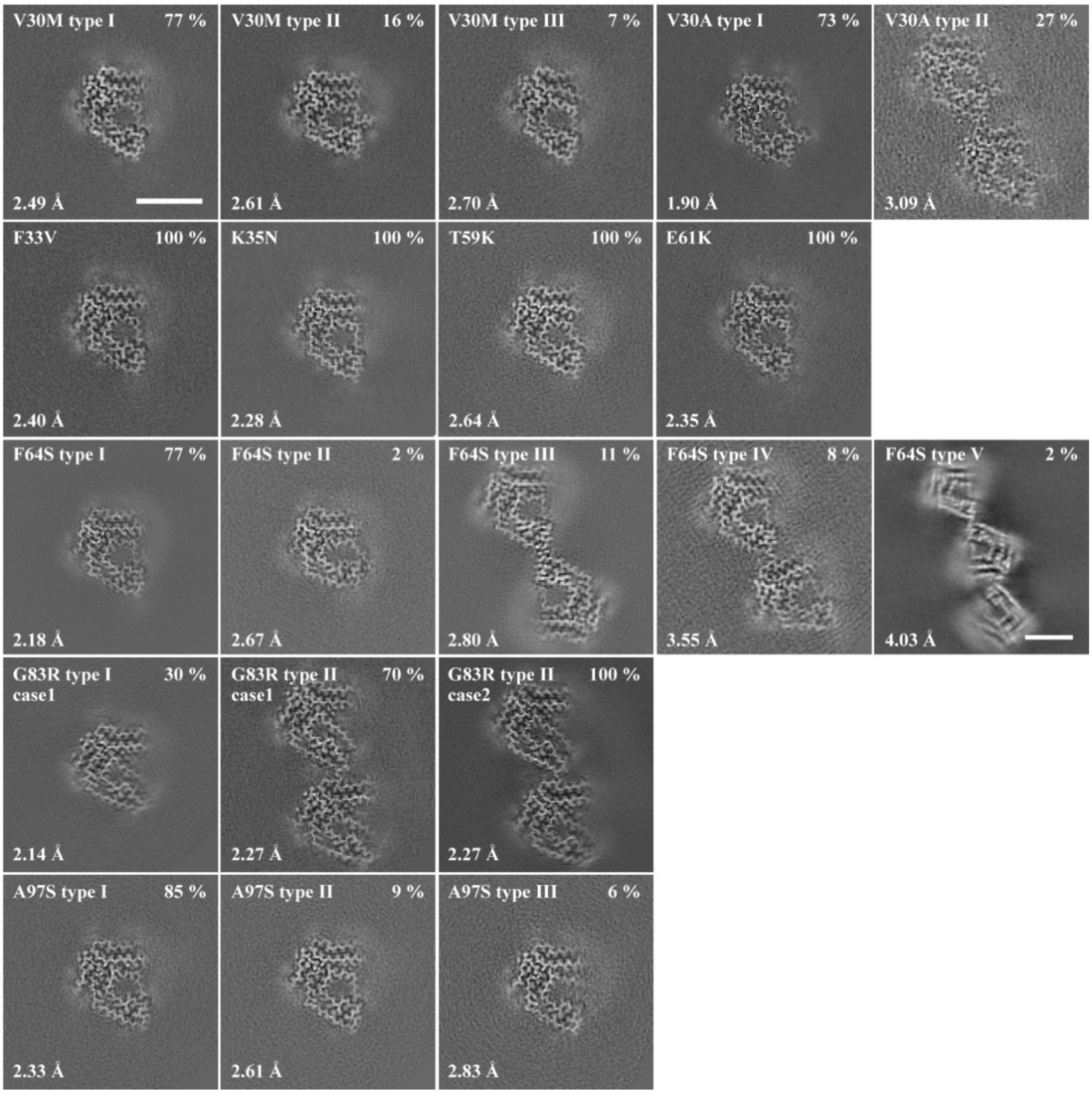

Supplementary Fig. 2 | Cryo-EM reconstructions.

Cross-sections through the cryo-EM reconstructions, perpendicular to the helical axis and with a projected thickness of approximately one rung, are shown for the gastrocnemius muscle tissue from patients with single-point mutations of V30M, V30A, F33V, K35N, T59K, E61K, G83R (n=2), and A97S, as well as the vitreous body from an individual with an F64S mutation. For each case, cryo-EM data were collected and processed from a single preparation. Structural types, percentages of each structural type, and resolutions (in Å) are indicated at the top left, top right, and bottom left, respectively. Scale bars, 5 nm. The scale bar shown for the ATTRv-V30M type I structure applies to all reconstructions, except for the ATTRv-F64S type V structure.

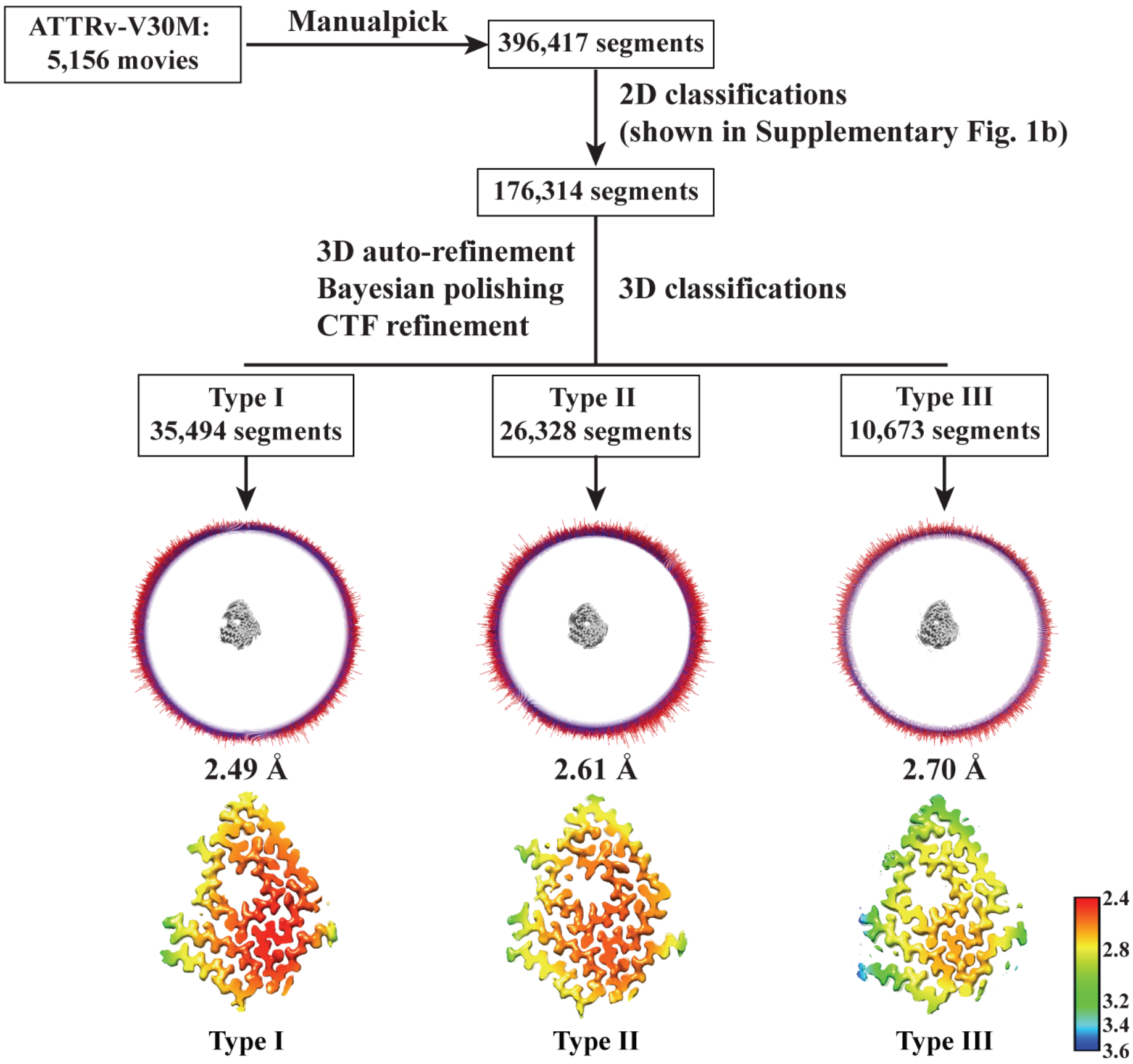

Supplementary Fig. 3 | Cryo-EM data processing of TTR fibrils.

Cryo-EM image-processing workflow for TTR filaments from an ATTRv-V30M patient, shown as a representative example. Particle angular distributions and local resolution maps are shown for the final reconstructions of ATTRv-V30M.

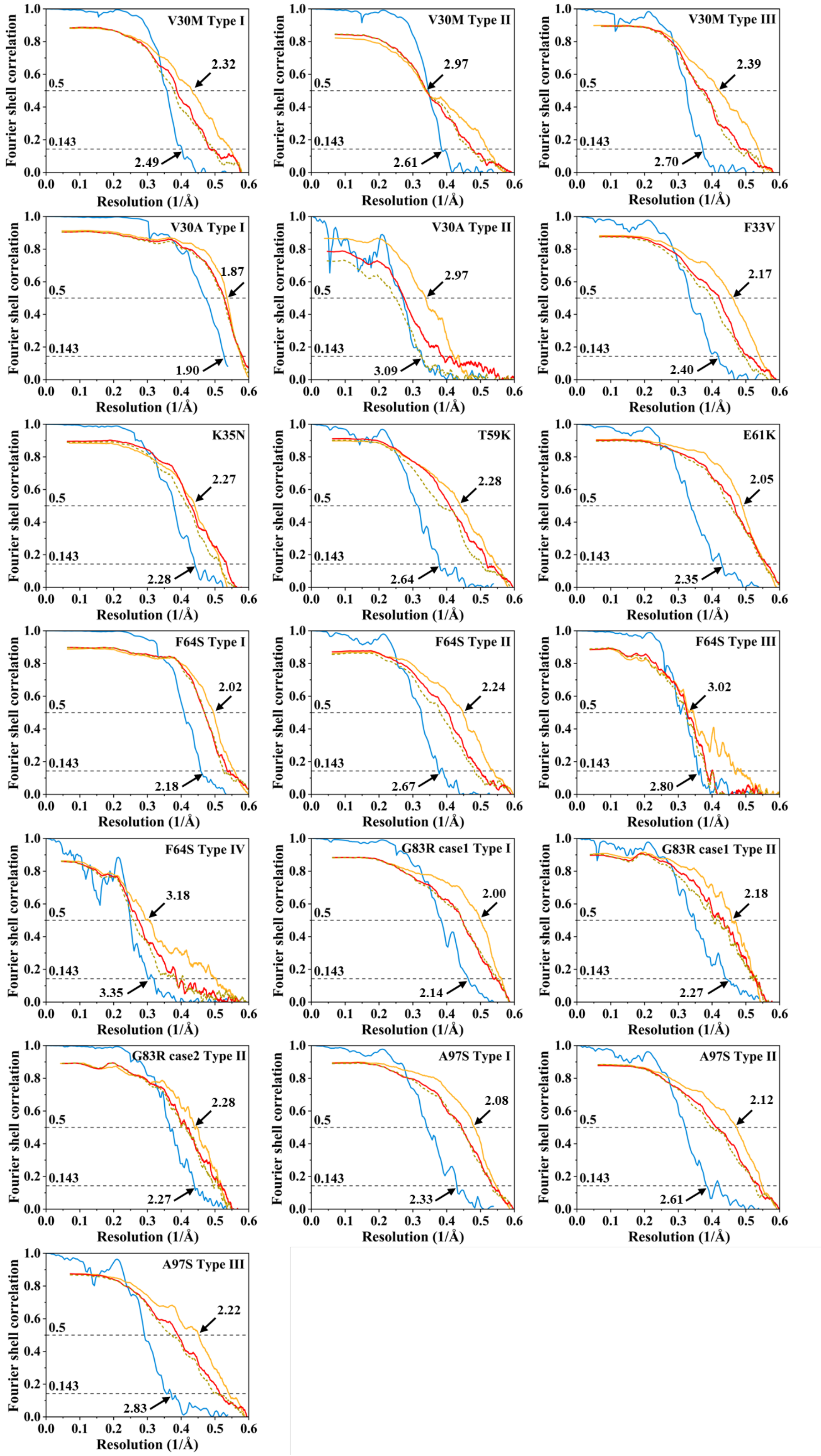

Supplementary Fig. 4 | Cryo-EM resolution estimates.

Fourier Shell Correlation (FSC) curves for the cryo-EM maps and atomic structures of TTR fibrils. FSC curves are shown for two independently refined cryo-EM half-maps (blue); for the final refined atomic model against the final cryo-EM map (yellow); for the atomic model refined in the first half-map against that half-map (red); and for the refined atomic model in the first half-map against the other half-map (dark yellow, dashed line). Source data are provided as a Source Data file.

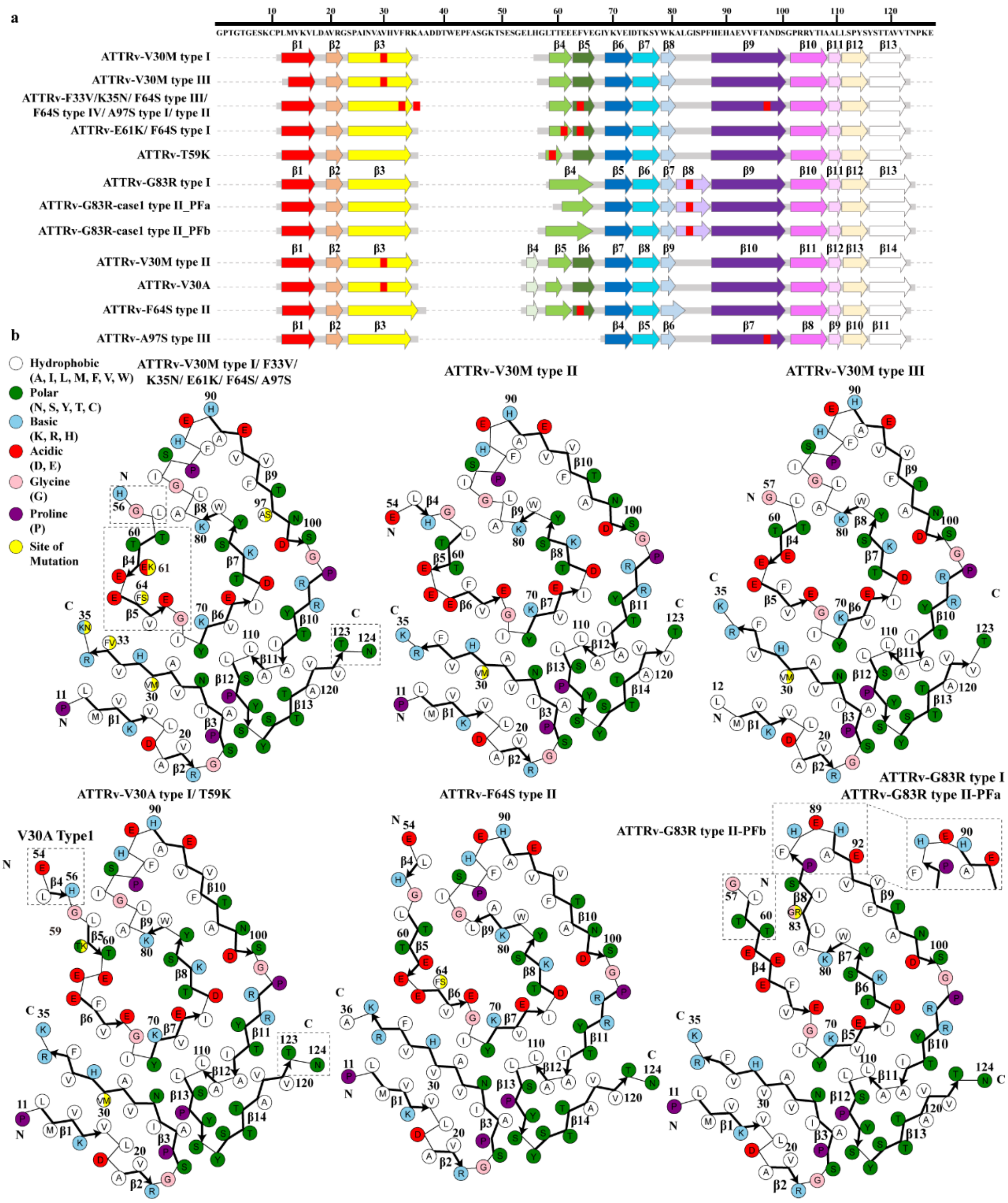

Supplementary Fig. 5 | Schematics of TTR fibril folds.

a, Amino acid sequence of wild-type TTR. Thick connecting lines with arrowheads indicate β-strands, with mutation sites highlighted. Residues indicated by grey dashed lines were not observed in the fibril cores. **b,** Schematics of TTR fibril folds. Negatively charged residues are in red, positively charged residues in blue, polar residues in green, non-polar residues in white, prolines in purple, glycines in pink, and mutant residues in yellow. Thick connecting lines with arrowheads indicate β-strands.

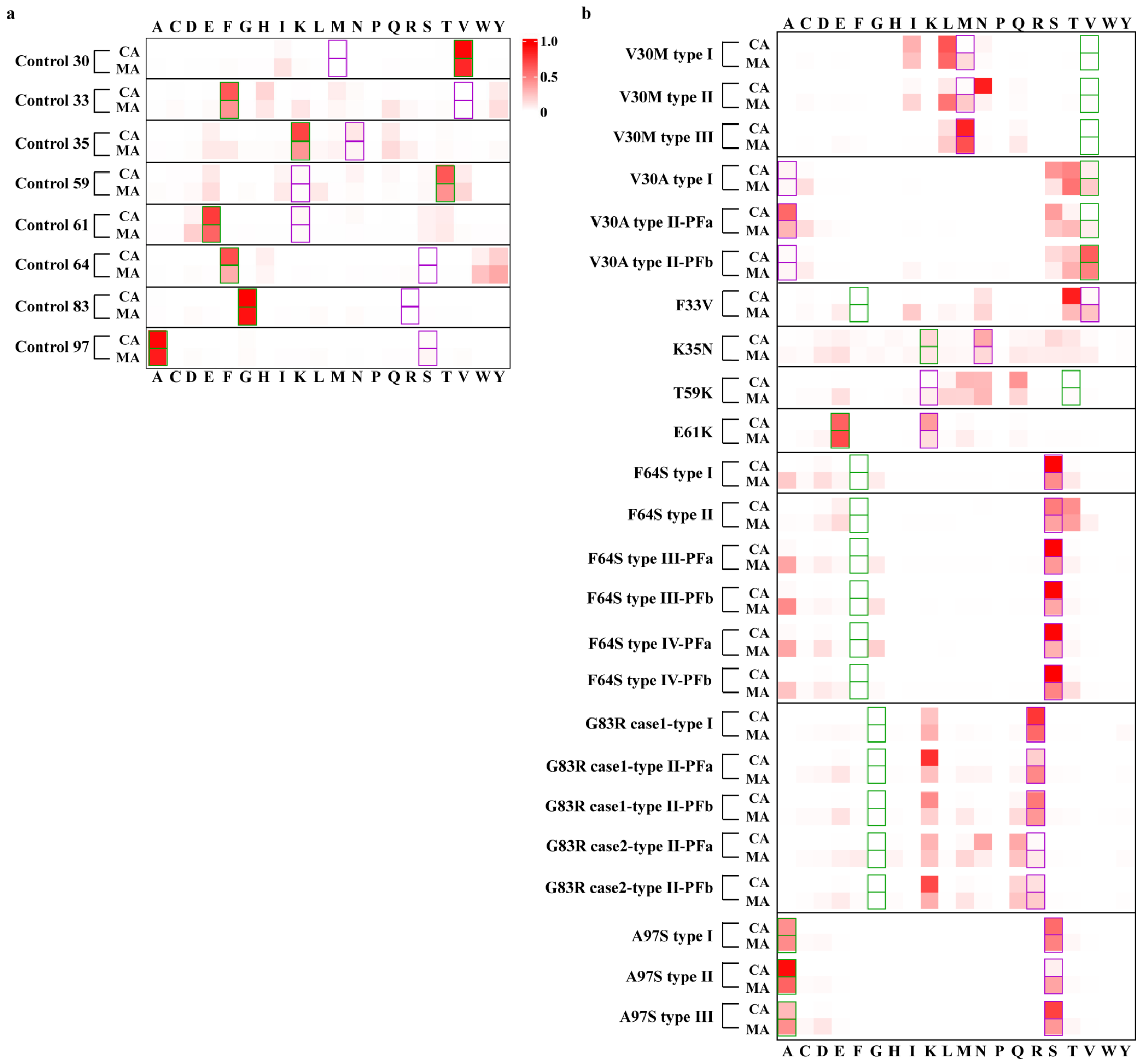

Supplementary Fig. 6 | Predicted amino acid probabilities at the mutant sites.

a, Average probabilities of all 20 amino acids at residues 30, 33, 35, 59, 61, 64, 83, and 97 in the control TTR fibril maps (Supplementary Data 3), predicted by CryoAtom (CA) and ModelAngelo (MA). Probabilities are represented using a color gradient from red (high probability, 1) to white (low probability, 0). **b,** Average probabilities of all 20 amino acids at the mutation sites (Supplementary Data 4), using the same color scheme as in (**a**).

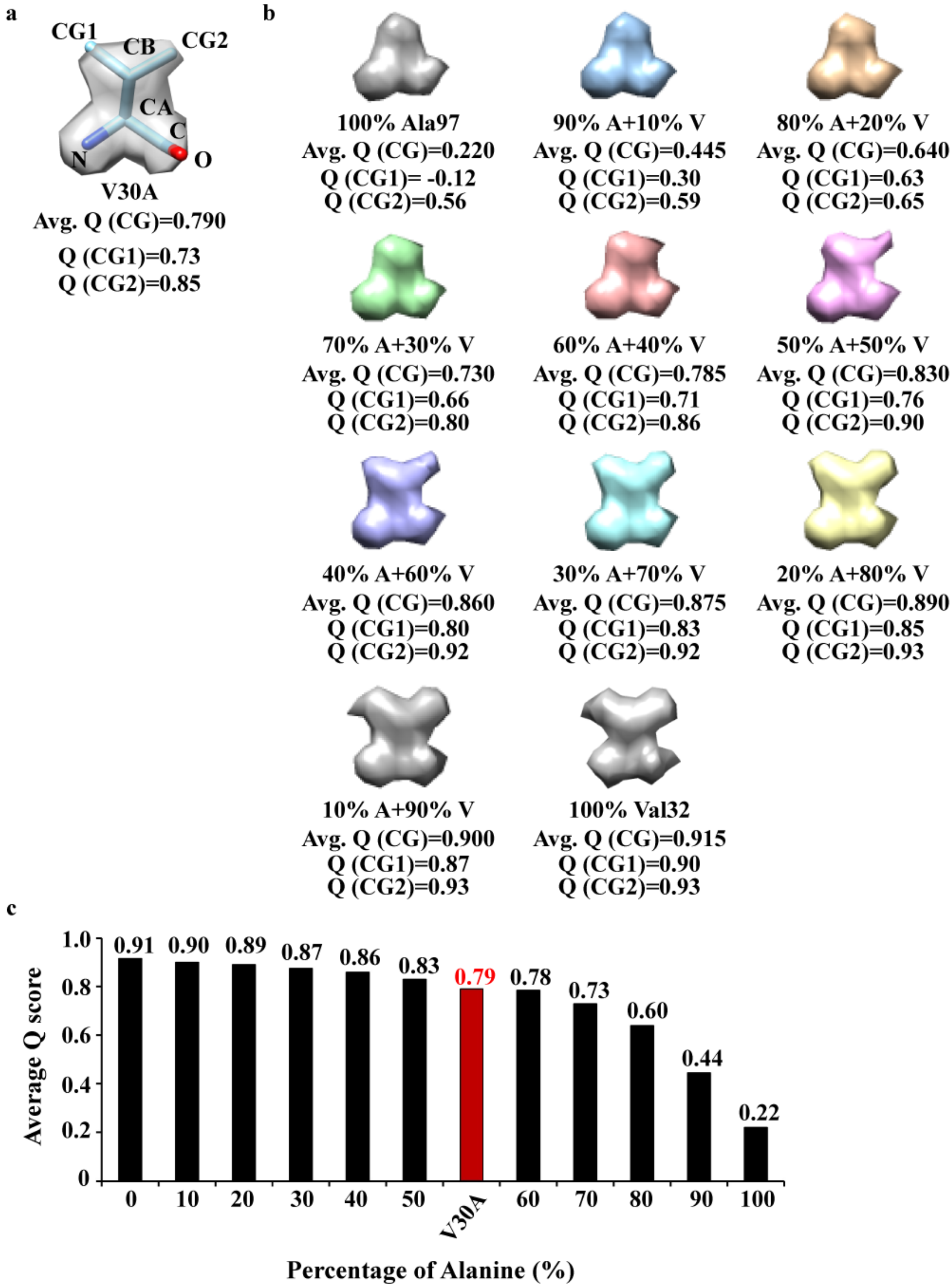

Supplementary Fig. 7 | Estimation of the alanine-to-valine ratio at residue 30 in the cryo-EM map of the ATTRv-V30A type I structure. **a,** Cryo-EM map of residue 30 of ATTRv-V30A type I structure, overlaid with a valine model. Q-scores for the γ-carbon atoms of valine (CG1 and CG2), along with their average, are shown. **b,** Cryo-EM maps of residues V32 and

A97 from the same map, and their averaged maps at varying mixing ratios. **c,** Histogram showing the average Q-scores of the γ-carbon atoms of valine for each residue map. The bar corresponding to residue 30 in the cryo-EM map of the ATTRv-V30A type I structure is highlighted in red. Source data are provided as a Source Data file.

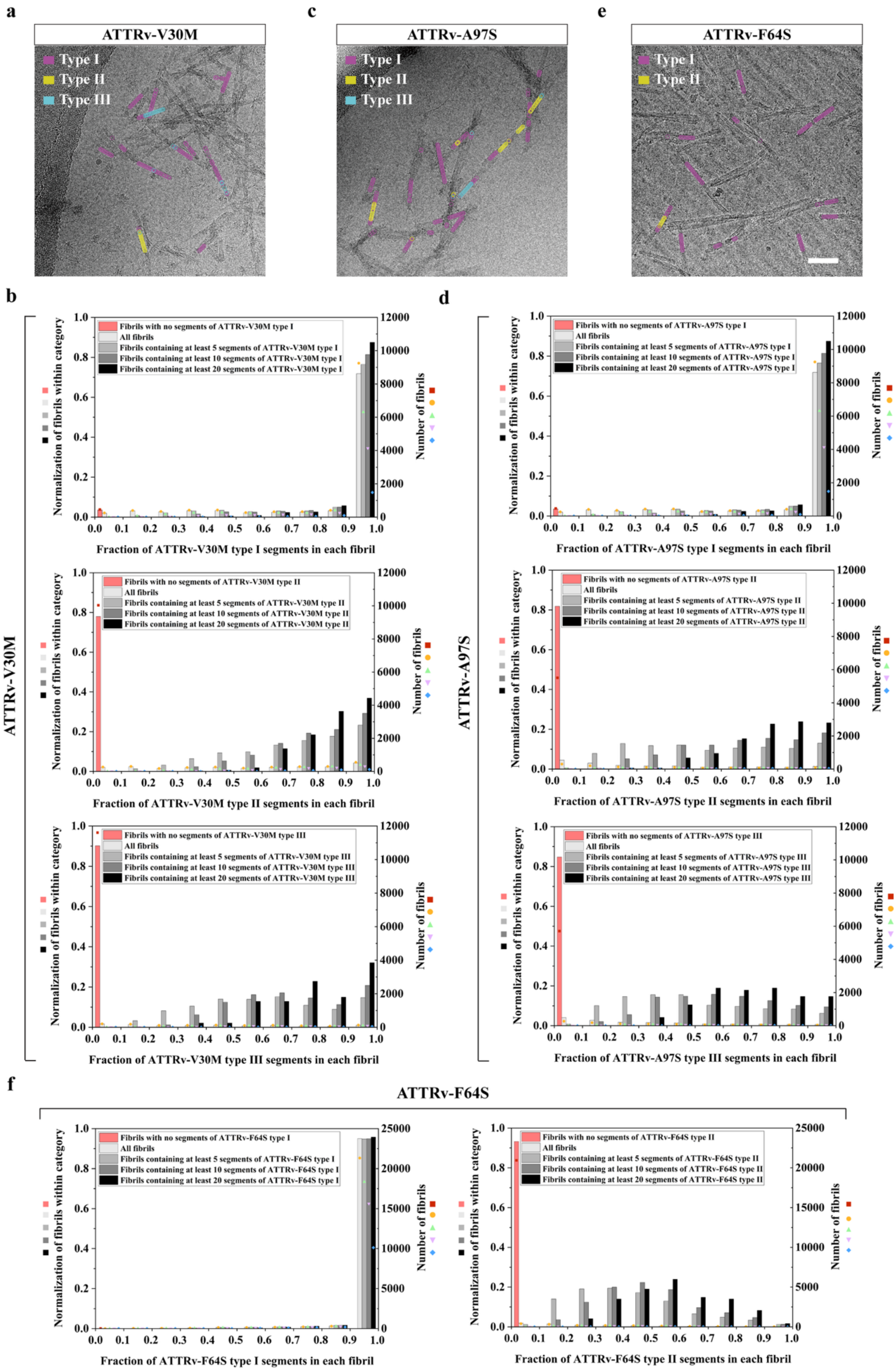

Supplementary Fig. 8 | Coexistence of distinct conformations within single fibrils.

a, c, e, Representative cryo-EM micrographs from patients with single-point mutations of V30M (**a**), A97S (**c**), and F64S (**e**), showing the locations of segments classified to each structural type by 3D classification. Scale bar, 50 nm. **b**, **d**, **f**, Histogram showing the fraction of segments assigned to each structural type in ATTRv-V30M (**b**), ATTRv-A97S (**d**) and ATTRv-F64S (**f**). Different thresholds were chosen for the minimum number of segments per fibril, resulting in four categories: all fibrils, fibrils with a minimum of 5, 10, or 20 segments of the analyzed class. Fibrils that do not contain any segments of the analyzed type are shown separately in red. Percentages were normalized to the total number of fibrils in each category. Colored points show the absolute number of fibrils in each category and group. Source data are provided as a Source Data file.

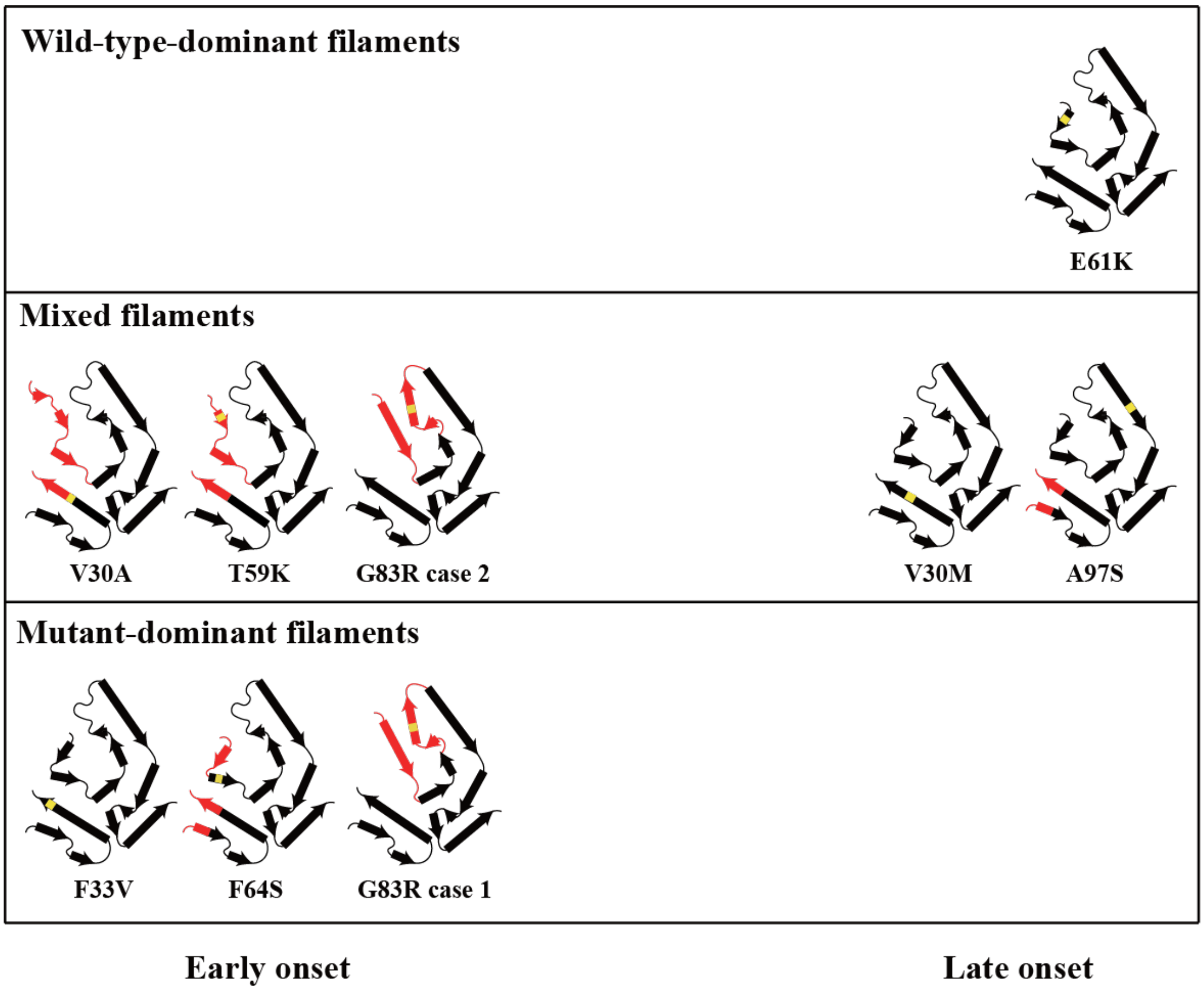

Supplementary Fig. 9 | Structural and compositional overview of patient-derived fibrils in relation to disease onset.

Rendered views of the secondary structure elements of representative ATTRv folds from each patient. Regions structurally identical to the ATTRwt fold are shown in black, and those with structural changes are shown in red. Mutation sites are highlighted in yellow.

Supplementary Table 1 | Cases of ATTRv amyloidosis.

No.	Mutation	Course (years)	Age at onset (years)	Coutinho stage at first visit	Family History	Initial manifestation	Phenotype
1	V30M	2.5	⩾50	I	+	Paresthesia	PN+C+CTS+Cough+AN
2	V30A	3	＜50	I	+	OH	AN+PN+C+Cough
3	F33V	2.5	＜50	I	+	Impotence	PN+C+AN+E+Cough
4	K35N	6	＜50	II	+	Painful paresthesia	PN+AN+C+E
5	T59K	6	＜50	I	+	Diarrhea	CTS+AN+C+E+Cough
6	E61K	3	⩾50	II	+	Impotence	PN+AN+C
7	F64S	2	＜50	NA	NA	Blurred vision	E
8	G83R	5	＜50	I	+	Blurred vision	PN+AN+C+E+H
9	G83R	5	＜50	I	+	Blurred vision	PN+E+AN+C+CNI
10	A97S	6	⩾50	II	+	CTS	PN+AN+C

OH, orthostatic hypotension; CTS, carpal tunnel syndrome; AN, autonomic neuropathy; C, cardiopathy; CNI, cranial nerves involvement; CNS, central nervous system; E, eye; H, hearing loss; PN, polyneuropathy; "+" means positive; "-" means negative.

Supplementary Table 2 | Structural alignment details.

Residues align	Atom pairs align	r.m.s.d.
8ADE 11-123 & ATTRv-V30M Type I 11-123	11-35, 56-123, 368	0.713
8ADE 11-123 & ATTRv-V30M Type II 11-123	11-35, 57-123, 368	1.736
8ADE 11-123 & ATTRv-V30M Type III 12-123	12-35, 57-123, 364	1.364
8ADE 11-123 & ATTRv-V30A Type I 11-123	11-35, 57-123, 368	1.443
8ADE 11-123 & ATTRv-F33V 11-123	11-35, 58-123, 364	0.800
8ADE 11-123 & ATTRv-K35N 11-123	11-35, 58-123, 364	0.774
8ADE 11-123 & ATTRv-T59K 11-123	11-35, 57-123, 368	1.455
8ADE 11-123 & ATTRv-E61K 11-123	11-35, 57-123, 368	0.758
8ADE 11-123 & ATTRv-F64S Type I 11-123	11-35, 57-123, 368	0.898
9HYW 11-123 & ATTRv-F64S Type I 11-123	11-35, 57-123, 368	0.649
8ADE 11-123 & ATTRv-F64S Type II 11-123	11-35, 57-123, 368	2.449
8ADE 11-123 & ATTRv-G83R Type I 11-123	11-35, 58-123, 364	3.067
8ADE 11-123 & ATTRv-G83R Type II-PFa 11-123	11-35, 60-123, 356	3.199
8ADE 11-123 & ATTRv-G83R Type II-PFb 11-123	11-35, 57-123, 368	3.446
8ADE 11-123 & ATTRv-A97S Type I 11-123	11-35, 58-123, 364	0.913
8ADE 11-123 & ATTRv-A97S Type II 11-123	11-35, 58-123, 364	0.668
8ADE 11-123 & ATTRv-A97S Type III 11-123	11-35, 68-123, 324	0.938
ATTRv-V30A Type I 11-123 & ATTRv-T59K 11-123	11-35, 57-123, 368	0.552
ATTRv-G83R case1 Type II 11-124 & ATTRv-G83R case2 Type II 11-124	PFa: 11-35, 60-124; PFb: 11-35, 57-124, 732	0.367

Supplementary Table 3 | Trypsin-digested TTR fragments detected by mass spectrometry from the vitreous body of a patient carrying an F64S mutation.

Peak No.	Experimental mass (Da)	Protein Variant	Assigned fragment	Theoretical mass (Da)
1	671.4	WT/F64S	16-21	671.4
2	703.4	WT/F64S	71-76	703.4
3	746.4	WT/F64S	10-15	746.4
4	762.4			762.4
5	832.4	WT/F64S	1-9	832.4
6	1365.7	WT/F64S	22-34	1365.8
7	1393.6	WT/F64S	36-48	1393.6
8	1415.8	WT/F64S	10-21	1415.8
9	1493.8	WT/F64S	22-35	1493.8
10	1521.7	WT/F64S	35-48	1521.7
11	2019.1	WT/F64S	16-34	2019.1
12	2209.1	WT/F64S	84-103	2209.1
13	2359.2	WT/F64S	105-126	2359.2
14	2394.1	F64S	49-70	2394.1
15	2454.1	WT		2454.1
16	2450.2	WT/F64S	81-103	2450.2
17	2488.3	WT/F64S	105-127	2488.3
18	2515.3	WT/F64S	104-126	2515.3
19	2644.4	WT/F64S	104-127	2644.4
20	3079.5	F64S	49-76	3079.5
21	3139.5	WT		3139.5
22	3643.7	F64S	49-80	3643.7

Supplementary Table 4 | Summary of structural types, relative abundances, and compositional classifications.

Onset type[a]	Patients	Structural types	WT controls: amino acid frequencies by CA/ MA (%)			Mutation sites: amino acid frequencies by CA/ MA (%)			Compositional classification (structure-level)	Relative abundances (%)	Compositional classification (patient-level)
			WT	Mutant	Others	WT	Mutant	Others			
Early-onset	F33V	I	90/70[b]	0/0	10/30	0/0	100/73[c]	0/27	Mutant	100	Mutant
	K35N	I	82/68[d]	9/2	9/30	7/13	93/50	0/37	-	100	-
	F64S	I	100/97[e]	0/0	0/3	0/0	100/100[f]	0/0	Mutant	77	Mutant
		II				0/0	100/97[f]	0/3	Mutant	2	
		III-PFa				0/0	100/100[f]	0/0	Mutant	11	
		III-PFb				0/0	100/100[f]	0/0			
		IV-PFa				0/0	100/100[f]	0/0	Mutant	8	
		IV-PFb				0/0	100/100	0/0			
		V				NA			NA		
	G83R case 1	I	100/100	0/0	0/0	0/0	100/100[d]	0/0	Mutant	30	Mutant
		II-PFa				0/0	100/97[d]	0/3	Mutant	70	
		II-PFb				0/0	100/77[d]	0/23	Mutant		
	G83R case 2	II-PFa				0/0	23/50[d]	77/50	Mixed	100	Mixed
		II-PFb				0/0	100/73[d]	0/27	Mutant		
	V30A	I	100/97	0/0	0/3	0/0	0/0	100/100	Mixed	73	Mixed
		II-PFa				0/0	87/50	13/50	Mixed	27	
		II-PFb				100/80	0/0	0/20	Wild-type		
	T59K	I	86/76[g]	2/0	12/24	0/0	0/3	100/97	Mixed	100	Mixed
Late-onset	V30M	I	100/97	0/0	0/3	0/0	0/0	100/100	Mixed	77	Mixed
		II				0/0	0/13	100/87	Mixed	16	
		III				0/0	100/90	0/10	Mutant	7	
	E61K	I	83/75	0/0	17/25	80/93	20/3	0/3	Wild-type	100	Wild-type
	A97S	I	100/100	0/0	0/0	23/43	77/57	0/0	Mixed	85	Mixed
		II				100/83	0/17	0/0	Wild-type	9	
		III				0/57	100/43	0/0	Mixed	6	

a. A threshold of 50 years was used to distinguish early-onset from late-onset cases. **b**. Including phenylalanine (F), histidine (H) and tyrosine (Y). **c**. Including threonine (T), valine (V) and isoleucine (I). **d**. Including lysine (K) and arginine (R). **e**. Including phenylalanine (F), tryptophan (W) and tyrosine (Y). **f**. Including serine (S), alanine (A), glycine (G) and threonine (T). **g**. Including threonine (T) and valine (V).
