# Supplementary material for: Cryo-EM structures of biopsy-derived TTR fibrils in hereditary transthyretin amyloidosis
Source: Nat Commun. 2026 Jul 24;17:9023. doi: 10.1038/s41467-026-75850-8 (PMC13507274; doi:10.1038/s41467-026-75850-8)
Supplement: Supplementary file 2 [file 41467_2026_75850_MOESM2_ESM.pdf]

Description of Additional Supplementary Files

File Name: Supplementary Data 1

Description: Cryo-EM data collection, refinement and validation.

File Name: Supplementary Data 2

Description: Frequencies of the most probable amino acids at 30 rungs of ATTR fibril maps, as predicted by CryoAtom (CA) and ModelAngelo (MA).

File Name: Supplementary Data 3

Description: Predicted amino acid probabilities in control maps using CryoAtom (CA) and ModelAngelo (MA).

File Name: Supplementary Data 4

Description: Predicted amino acid probabilities at mutation sites in ATTRv fibrils using CryoAtom (CA) and ModelAngelo (MA).
