# Supplementary material for: Cryo-EM structures of biopsy-derived TTR fibrils in hereditary transthyretin amyloidosis
Source: Nat Commun. 2026 Jul 24;17:9023. doi: 10.1038/s41467-026-75850-8 (PMC13507274; doi:10.1038/s41467-026-75850-8)
Supplement: Supplementary file 3 [file 41467_2026_75850_MOESM7_ESM.pdf]

nature portfolio

Corresponding author(s): Yang Shi, Lingchao Meng, Wei Chi

Last updated by author(s): Jul 9, 2026

Reporting Summary

Nature Portfolio wishes to improve the reproducibility of the work that we publish. This form provides structure for consistency and transparency in reporting. For further information on Nature Portfolio policies, see our Editorial Policies and the Editorial Policy Checklist.

Statistics

For all statistical analyses, confirm that the following items are present in the figure legend, table legend, main text, or Methods section.

n/a	Confirmed	
☐	☒	The exact sample size (*n*) for each experimental group/condition, given as a discrete number and unit of measurement
☐	☒	A statement on whether measurements were taken from distinct samples or whether the same sample was measured repeatedly
☒	☐	The statistical test(s) used AND whether they are one- or two-sided<br>*Only common tests should be described solely by name; describe more complex techniques in the Methods section.*
☒	☐	A description of all covariates tested
☒	☐	A description of any assumptions or corrections, such as tests of normality and adjustment for multiple comparisons
☐	☒	A full description of the statistical parameters including central tendency (e.g. means) or other basic estimates (e.g. regression coefficient) AND variation (e.g. standard deviation) or associated estimates of uncertainty (e.g. confidence intervals)
☒	☐	For null hypothesis testing, the test statistic (e.g. *F*, *t*, *r*) with confidence intervals, effect sizes, degrees of freedom and *P* value noted<br>*Give P values as exact values whenever suitable.*
☒	☐	For Bayesian analysis, information on the choice of priors and Markov chain Monte Carlo settings
☒	☐	For hierarchical and complex designs, identification of the appropriate level for tests and full reporting of outcomes
☒	☐	Estimates of effect sizes (e.g. Cohen's *d*, Pearson's *r*), indicating how they were calculated

Our web collection on statistics for biologists contains articles on many of the points above.

Software and code

Policy information about availability of computer code

Data collection	EPU (v3.8)
Data analysis	RELION (v5.0), CTFFIND (v4.1), Coot (v0.9.8), PHENIX (v1.21-5207-000), PyMOL (v3.1.1), ChimeraX (v1.7.1), Skyline (26.1), CryoAtom (1.0.0), ModelAngelo (1.0.10)

For manuscripts utilizing custom algorithms or software that are central to the research but not yet described in published literature, software must be made available to editors and reviewers. We strongly encourage code deposition in a community repository (e.g. GitHub). See the Nature Portfolio guidelines for submitting code & software for further information.

Data

Policy information about availability of data

All manuscripts must include a data availability statement. This statement should provide the following information, where applicable:

- Accession codes, unique identifiers, or web links for publicly available datasets
- A description of any restrictions on data availability
- For clinical datasets or third party data, please ensure that the statement adheres to our policy

Cryo-EM maps have been deposited in the Electron Microscopy Data Bank (EMDB) under accession numbers EMD-65779 for ATTRv-V30M Type I, EMD-65780 for ATTRv-V30M Type II, EMD-65781 for ATTRv-V30M Type III, EMD-65794 for ATTRv-V30A Type I, EMD-65795 for ATTRv-V30A Type II, EMD-65797 for ATTRv-F33V, EMD-65796 for ATTRv-K35N, EMD-65790 for ATTRv-T59K, EMD-65789 for ATTRv-E61K, EMD-65782 for ATTRv-F64S Type I, EMD-65783 for ATTRv-F64S Type II,

EMD-65784 for ATTRv-F64S Type III, EMD-65785 for ATTRv-F64S Type IV, EMD-65792 for ATTRv-G83R case1 Type I, EMD-65793 for ATTRv-G83R case1 Type II, EMD-65791 for ATTRv-G83R case2 Type II, EMD-65786 for ATTRv-A97S Type I, EMD-65787 for ATTRv-A97S Type II, EMD-65788 for ATTRv-A97S Type III. Corresponding refined atomic models have been deposited in the Protein Data Bank (PDB) under accession numbers 9W9J for ATTRv-V30M Type I, 9W9K for ATTRv-V30M Type II, 9W9L for ATTRv-V30M Type III, 9W9Z for ATTRv-V30A Type I, 9WA0 for ATTRv-V30A Type II, 9WA2 for ATTRv-F33V, 9WA1 for ATTRv-K35N, 9W9V for ATTRv-T59K, 9W9U for ATTRv-E61K, 9W9N for ATTRv-F64S Type I, 9W9O for ATTRv-F64S Type II, 9W9P for ATTRv-F64S Type III, 9W9Q for ATTRv-F64S Type IV, 9W9X for ATTRv-G83R case1 Type I, 9W9Y for ATTRv-G83R case1 Type II, 9W9W for ATTRv-G83R case2 Type II, 9W9R for ATTRv-A97S Type I, 9W9S for ATTRv-A97S Type II, 9W9T for ATTRv-A97S Type III. The mass spectrometry proteomics data have been deposited to the ProteomeXchange Consortium (https://proteomecentral.proteomexchange.org) via the iProX partner repository with the dataset identifier PXD070068. The genotyping sequencing data of mutations in TTR patients have been deposited in the Genome Sequence Archive in National Genomics Data Center, China National Center for Bioinformation / Beijing Institute of Genomics, Chinese Academy of Sciences (GSA-Human: HRA019078). Source Data are provided with this paper. Unless otherwise stated, all data supporting the results of this study can be found in the article, supplementary, and source data files.

Research involving human participants, their data, or biological material

Policy information about studies with human participants or human data. See also policy information about sex, gender (identity/presentation), and sexual orientation and race, ethnicity and racism.

Reporting on sex and gender	Among the ten patients, one was female and nine were male. Sex was not considered as a biological variable because of limited tissue availability.
Reporting on race, ethnicity, or other socially relevant groupings	All participants in this study were of Chinese descent.
Population characteristics	See Extended Data Table 1.
Recruitment	See the Methods section. Samples were obtained from patients with ATTRv amyloidosis according to tissue availability.
Ethics oversight	The use of samples in this study was approved by the ethical review process at the Peking University First Hospital (2019-181) and the First Affiliated Hospital of Guangzhou Medical University (2024-IIT-16). Informed consent was obtained from all patients.

Note that full information on the approval of the study protocol must also be provided in the manuscript.

Field-specific reporting

Please select the one below that is the best fit for your research. If you are not sure, read the appropriate sections before making your selection.

☒ Life sciences ☐ Behavioural & social sciences ☐ Ecological, evolutionary & environmental sciences

For a reference copy of the document with all sections, see nature.com/documents/nr-reporting-summary-flat.pdf

Life sciences study design

All studies must disclose on these points even when the disclosure is negative.

Sample size	Gastrocnemius muscle samples were collected from patients with ATTR-V30M (n=1), ATTR-A97S (n=1), ATTR-E61K (n=1), ATTR-T59K (n=1), ATTR-G83R (n=2), ATTR-V30A (n=1), ATTR-K35N (n=1), and ATTR-F33V (n=1), and vitreous body from ATTR-F64S (n=1). Samples were chosen based on tissue availability.
Data exclusions	Pre-established common image classification procedures (S.H.W. Scheres, J. Struc. Biol. 180: 519-530, (2012)) were employed to select particle images with the highest resolution content in the cryo-EM reconstruction process. Details of the number of selected images are given in Supplementary Data 1.
Replication	For ATTRv-G83R, we examined two biological replicates, and representative data are shown as described in the main text. For the other mutations, only one case was analyzed for each due to sample availability.
Randomization	As this study was not designed to compare different treatment groups, randomization was not applicable.
Blinding	The investigators were not blinded to allocation during experiments and outcome assessment. The perceived risk of detection/performance bias was deemed negligible.

Reporting for specific materials, systems and methods

We require information from authors about some types of materials, experimental systems and methods used in many studies. Here, indicate whether each material, system or method listed is relevant to your study. If you are not sure if a list item applies to your research, read the appropriate section before selecting a response.

Materials & experimental systems

n/a	Involved in the study
☒	☐ Antibodies
☒	☐ Eukaryotic cell lines
☒	☐ Palaeontology and archaeology
☒	☐ Animals and other organisms
☒	☐ Clinical data
☒	☐ Dual use research of concern
☒	☐ Plants

Methods

n/a	Involved in the study
☒	☐ ChIP-seq
☒	☐ Flow cytometry
☒	☐ MRI-based neuroimaging

Plants

Seed stocks	Not relevant to study.
Novel plant genotypes	Not relevant to study.
Authentication	Not relevant to study.
