# Supplementary material for: Cryo-EM structures of biopsy-derived TTR fibrils in hereditary transthyretin amyloidosis
Source: Nat Commun. 2026 Jul 24;17:9023. doi: 10.1038/s41467-026-75850-8 (PMC13507274; doi:10.1038/s41467-026-75850-8)
Supplement: Supplementary file 4 [file 41467_2026_75850_MOESM8_ESM.pdf]

nature portfolio

Peer Review File

Cryo-EM Structures of Biopsy-derived TTR Fibrils in Hereditary Transthyretin Amyloidosis

Corresponding Author: Dr Yang Shi

This file contains all reviewer reports in order by version, followed by all author rebuttals in order by version.

Version 0:

Reviewer comments:

Reviewer #1

(Remarks to the Author)
This manuscript reports very interesting research, the data are of very high quality, no technical flaws were detected, the text is very well written. I strongly support publication provided that the following amendments are included.

Major:
1.
The text frequently refers to the different reconstructions from one patient as different “filaments”. This is not necessarily true. It is also possible that these reconstructions represent only different structural forms of the fibril protein that occur at different positions of the same filament. See for example DOI: 10.1038/s41467-021-21126-2 . This reference contains also a method to check whether the different forms occur as part of the same fibril or whether they represent truly different filaments. This type of analysis should be done here as well. Moreover, I generally recommend to refer to the different structures from one case indeed as “structures” and not as filaments, in particular if it should turn out through the requested analysis that they can co-occur in the same filament.

2.
The manuscript should contain in the SI raw cryo-EM images from each case with the different types of filaments marked. Some fibrils must be different, for example the cross-sectional dimers.

3.
Extended data Fig. 7: While this subdivision of structures it is interesting, it is somewhat unclear how the structures are assigned to the different groups. E.G., why is K35N mutant dominated and V30M mixed and not vice versa? How are variations in the MA/CF probabilities for different structures of the same mutant dealt with? Perhaps this requires an additional figure/table.

Minor:

1.
The introduction claims that previous amyloid structural studies commonly relied on post mortem tissue. That may be true for analysis of neurodegenerative amyloids, while many studies on systemic amyloidosis – the kind of diseases relevant to this work – were done with biopsies or explanted organs, mainly hearts, due to cardiac surgery.

2.
Line 126: The N-terminal part of the structure is misclassified as a “hairpin”. A hairpin contains two antiparallel strands that from one common sheet; i.e. they are backbone hydrogen bonded to one another. The structure of the fibril is correctly called a “beta-arch”.

3.
Line 319: It is misleading to refer to this structure as “to explore the influence of the ocular environment” is not correct and misleading. Without a F64S sample from another tissue it cannot be discriminated whether any changes are mutation- or tissue-dependent. Hence, this aim should be deleted.
4.

Line 382: the statement on the tilt is hard to follow from the figure. I recommend to highlight it with an arrow in Fig. 5b.

5.
Fig. 2b: The structures cannot be easily discriminated. In particular the WT structure should be coloured in a way that cannot be confused with G83R case I type II-PFb.

6.
Extended Data Fig. 3: The axis labels and other writings are too small.

7.
Fig. 3 j-l: it is only indirectly clear from the legend, which structures are analysed here. Please describe in the figure caption.

Reviewer #2

(Remarks to the Author)
By prior agreement, I evaluated all parts of the manuscript except for the cryo-electron microscopy (cryo-EM) data. Overall, the article is well written, logically structured, and scientifically engaging. Nevertheless, I have several substantive concerns regarding the characterization of the source material and the presentation of some data.
Major comments:
1. Before proceeding to structural analysis, the authors aimed to demonstrate that the examined tissue samples contained transthyretin (TTR) amyloid inclusions. To achieve this, it is essential to show colocalization of TTR with Congo red. Extended Data Fig. 1a presents Congo red staining and TTR immunostaining; however, the authors did not attempt to assess colocalization. The upper panels show regions stained with Congo red, while the lower panels show the regions stained with anti-TTR antibodies. Apparent colocalization is visible in only three of the eight images. As presented, these results do not allow the conclusion that the studied samples contain TTR amyloid deposits. In their current form, the data are inconclusive and unsuitable for publication.
Given that the central focus of the article is the cryo-EM analysis of TTR fibrils, I can offer the authors two options for solving this problem:
• Provide high-quality images clearly demonstrating colocalization of TTR with Congo red, or
• Remove the immunohistochemistry and immunoblotting data entirely. Keep only the data presented in section "b" of Extended Data Fig. 1.
2. The authors discuss whether the examined mutations influence filament structure. However, the manuscript provides almost no discussion of how the substituted amino acids differ from the wild-type residues in terms of charge, hydrophobicity, steric properties, or other physicochemical parameters. These differences may plausibly impact filament architecture. I strongly encourage the authors to expand the discussion to include these considerations.
Minor comments:
1. Mutation data availability
Sequencing data for all mutations should be deposited in an accessible public database.
2. Extended Data Fig. 1
The caption for panel “c” should be expanded and clarified if this result is retained in the final version of the manuscript.
3. Lines 548–553:
The authors should specify the secondary antibodies used, including vendor information and working dilutions.
4. Mass spectrometry data
Although the mass spectrometry (MS) results appear technically sound, the rationale for performing MS exclusively on the F64S variant is unclear. The manuscript does not explain the purpose of the MS experiment nor how the results complement or validate the cryo-EM findings. This requires clarification.
In general, the manuscript can be accepted for publication after corrections to the comments.

Version 1:

Reviewer comments:

Reviewer #1

(Remarks to the Author)
The authors could address several of the issues raised in the last evaluations. However, three problems remain and are not dealt with in an appropriate fashion.

1. The use of the term filament is still inappropriate.
In their rebuttal letter, the authors claim to have substituted the term “filament” with “structure” wherever appropriate, but I cannot see that this was done - except in a few cases. The manuscript is still full of the term “filament”, and this is problematic for two reasons: First, the authors seem to uniformly write “amyloid filament” when meaning “amyloid fibril”. This gives the impression that they do not mean “amyloid fibril” but something else, perhaps a substructure of an amyloid fibril, such as a protofilament. For clarity, the phrase “amyloid fibril” should be used wherever appropriate; that is, in most cases. Second, the term “filament” implies a linear single object. Following my request for additional analysis (new Fig. 7), the authors now admit that many of their reconstructions do not represent discrete filaments but different structures within the same filament. In these cases, reconstructions need to be referred to as “reconstructions” or “structures” but not as

“filaments”.

2. The assignment of structures in SI Fig. 8 or the revised manuscript.
The assignment of structures could neither be followed in the previous version nor in the revised manuscript. I now explicitly require a separate methods section describing the assignment. This section should state the age cut off values for assignment to early and late onset and the parameters and cut off values used for the assignment into “wildtype dominated”, “mixed” or “mutant dominated”. In addition, I request a table listing for each mutant and reconstruction the age of onset, the assignment towards early and late onset, and, for the assignment into “wildtype dominated”, “mixed” or “mutant dominated”, the relevant parameter values per reconstruction, and the resulting assignment into “wildtype dominated”, “mixed” or “mutant dominated”.

3. The informational content of Fig. 2b
The colour coding has changed but the blue structures in the two close ups are different from one another and they are also different from the overview image. Therefore, the informational content of this image is still unclear.

Reviewer #2

(Remarks to the Author)
The authors responded to all my questions and comments. In my opinion, the manuscript is suitable for publication.

Version 2:

Reviewer comments:

Reviewer #1

(Remarks to the Author)
The authors have adequately dealt with my comments in most cases. In particular, they now explain how they subdivided the reconstructions into different categories. They also removed the word filament in most cases as required, although they simply substituted it with fibril in almost all of the cases.

However, I asked to use the term “structure” instead of filament if the possibility cannot be excluded that two reconstructions show a different regions/segment of the same fibril. This is the case for several reconstructions where the authors now show in their new SI Fig. 7 that the reconstructions represent different segments of the same fibril but not necessarily different fibrils.

Open Access This Peer Review File is licensed under a Creative Commons Attribution 4.0 International License, which permits use, sharing, adaptation, distribution and reproduction in any medium or format, as long as you give appropriate credit to the original author(s) and the source, provide a link to the Creative Commons license, and indicate if changes were

made.
In cases where reviewers are anonymous, credit should be given to 'Anonymous Referee' and the source.
The images or other third party material in this Peer Review File are included in the article's Creative Commons license, unless indicated otherwise in a credit line to the material. If material is not included in the article's Creative Commons license and your intended use is not permitted by statutory regulation or exceeds the permitted use, you will need to obtain permission directly from the copyright holder.
To view a copy of this license, visit https://creativecommons.org/licenses/by/4.0/

We thank the reviewers for their constructive and helpful comments, which we feel have contributed to an improved manuscript. In addition to addressing the reviewers' comments, a recent study on skin-derived ATTRv-F64S filaments has been published (PMID: 41402329), and we have now cited it in the manuscript (lines 333-334, Fig. 4a). The software CryFold (CF), which we used for amino acid prediction, has been officially renamed CryoAtom (CA) as reported in a recent publication (PMID: 41238963). The software name has been updated throughout the manuscript.

Reviewer #1 (Remarks to the Author):

This manuscript reports very interesting research, the data are of very high quality, no technical flaws were detected, the text is very well written. I strongly support publication provided that the following amendments are included.

Major:

1.

The text frequently refers to the different reconstructions from one patient as different "filaments". This is not necessarily true. It is also possible that these reconstructions represent only different structural forms of the fibril protein that occur at different positions of the same filament. See for example DOI: 10.1038/s41467-021-21126-2 . This reference contains also a method to check whether the different forms occur as part of the same fibril or whether they represent truly different filaments. This type of analysis should be done here as well. Moreover, I generally recommend to refer to the different structures from one case indeed as "structures" and not as filaments, in particular if it should turn out through the requested analysis that they can co-occur in the same filament.

We thank the reviewer for this important suggestion. Following the approach suggested by the reviewer, we have now performed a similar analysis to examine whether different structural types can coexist within individual filaments. This analysis has been included in the revised manuscript (lines 420-436; Supplementary Fig. 7) and shows that the different reconstructions obtained from one patient do not necessarily arise from distinct filaments. Therefore, we have replaced the term "filament" with "structure" when referring to different reconstructions from the same case throughout the manuscript, to avoid implying that they necessarily correspond to distinct filaments.

2.

The manuscript should contain in the SI raw cryo-EM images from each case with the different types of filaments marked. Some fibrils must be

different, for example the cross-sectional dimers.

In the original manuscript, Supplementary Fig. 1b shows representative cryo-EM micrographs of TTR filaments from all cases, with single-, double-, and triple-protofilament assemblies labeled. The corresponding 2D class averages are shown in the lower left corner of each micrograph. The different single-protofilament assemblies can hardly be distinguished in either the raw micrographs or the 2D class averages. They can only be resolved by 3D classification, as shown in Supplementary Fig. 7.

3.
Extended data Fig. 7: While this subdivision of structures it is interesting, it is somewhat unclear how the structures are assigned to the different groups. E.G., why is K35N mutant dominated and V30M mixed and not vice versa? How are variations in the MA/CF probabilities for different structures of the same mutant dealt with? Perhaps this requires an additional figure/table.

The WT/mutant TTR composition at the patient level was determined based on the structure-level composition of each filament type and its relative abundance in the patient tissue. Specifically, each structure was classified into one of three categories—wild-type–dominated, mutant-dominated, or mixed—based on its MA/CA probabilities. If only a single structure was identified in a given patient, the composition of that structure was taken to represent the overall composition of the patient. When multiple structural types were present in one patient and were classified into different categories, the overall composition of the patient was defined by the major structural type, i.e., the one comprising a substantially greater number of segments. In our study, we did not encounter highly complex cases, and this approach was applicable to all cases.

For example, in the ATTRv-K35N patient, only a mutant-dominated structural type was detected, and the deposits in this case were therefore classified as mutant-dominated. In contrast, tissues from the ATTRv-V30M patient contained three structural types. Although the type III structure was mutant-dominated, it represented only a minor population (7%). The majority of segments (93%) corresponded to type I and type II structures, which were classified as mixed. Accordingly, the patient-level deposits were classified as mixed.

The WT/mutant TTR composition at the patient level, previously described at the end of each Results subsection, is now summarized in Supplementary Table 8
Minor:

1.
The introduction claims that previous amyloid structural studies commonly relied on post mortem tissue. That may be true for analysis of neurodegenerative amyloids, while many studies on systemic amyloidosis – the kind of diseases relevant to this work – were done with biopsies or explanted organs, mainly hearts, due to cardiac surgery.

We agree with the reviewer that many structural studies of systemic amyloidosis have been performed using biopsy samples or explanted organs, particularly cardiac tissue obtained during surgery. To avoid overgeneralization, we have therefore removed the corresponding statement "*Previously, amyloid structural studies have commonly relied on post-mortem tissues.*" from the Introduction in the revised manuscript.

2.
Line 126: The N-terminal part of the structure is misclassified as a "hairpin". A hairpin contains two antiparallel strands that from one common sheet; i.e. they are backbone hydrogen bonded to one another. The structure of the fibril is correctly called a "beta-arch".

As suggested, we have corrected the terminology in the revised manuscript and now refer to the N-terminal motif as a "β-arch" instead of a "hairpin".

3.
Line 319: It is misleading to refer to this structure as "to explore the influence of the ocular environment" is not correct and misleading. Without a F64S sample from another tissue it cannot be discriminated whether any changes are mutation- or tissue-dependent. Hence, this aim should be deleted.

As suggested, we have removed the aim in the revised manuscript.

4.
Line 382: the statement on the tilt is hard to follow from the figure. I recommend to highlight it with an arrow in Fig. 5b.

As suggested, arrows have been added to Fig. 5b to highlight the tilt.

5.
Fig. 2b: The structures cannot be easily discriminated. In particular the WT structure should be coloured in a way that cannot be confused with G83R case I type II-PFb.

We have revised the color scheme in Fig. 2b to improve discrimination

between the structures.

6.
Extended Data Fig. 3: The axis labels and other writings are too small.

We have improved the readability of Supplementary Fig. 3 in the revised manuscript.

7.
Fig. 3 j-l: it is only indirectly clear from the legend, which structures are analysed here. Please describe in the figure caption.

The more detailed description has now been included in the figure caption: *"In panels j-l, frequencies of the only structure identified in each case are shown: ATTRv-F33V (j), ATTRv-K35N (k), ATTRv-T59K (l)."* To improve the readability of such panels, we have labeled the predictions derived from maps without a mutation at the site of interest as "WT control" and those containing the mutation as "ATTRv-(mutant type)."

Reviewer #2 (Remarks to the Author):

By prior agreement, I evaluated all parts of the manuscript except for the cryo-electron microscopy (cryo-EM) data. Overall, the article is well written, logically structured, and scientifically engaging. Nevertheless, I have several substantive concerns regarding the characterization of the source material and the presentation of some data.
Major comments:
1. Before proceeding to structural analysis, the authors aimed to demonstrate that the examined tissue samples contained transthyretin (TTR) amyloid inclusions. To achieve this, it is essential to show colocalization of TTR with Congo red. Extended Data Fig. 1a presents Congo red staining and TTR immunostaining; however, the authors did not attempt to assess colocalization. The upper panels show regions stained with Congo red, while the lower panels show the regions stained with anti-TTR antibodies. Apparent colocalization is visible in only three of the eight images. As presented, these results do not allow the conclusion that the studied samples contain TTR amyloid deposits. In their current form, the data are inconclusive and unsuitable for publication.
Given that the central focus of the article is the cryo-EM analysis of TTR fibrils, I can offer the authors two options for solving this problem:
• Provide high-quality images clearly demonstrating colocalization of TTR with Congo red, or
• Remove the immunohistochemistry and immunoblotting data entirely. Keep only the data presented in section "b" of Extended Data Fig. 1.

We fully agree with the reviewer's concern. Ideally, colocalization of TTR immunoreactivity with Congo red–positive deposits should be demonstrated to confirm the presence of TTR amyloid inclusions in the examined tissues. We carefully re-examined the remaining samples and all available data. However, due to limited tissue availability, we are unable to provide matched TTR immunostaining and Congo red staining for the five cases.

Given this limitation, and as suggested by the reviewer, we have removed the immunohistochemistry and immunoblotting data from Supplementary Fig. 1 in the revised manuscript, and the revised manuscript now focuses on the cryo-EM analysis of TTR fibrils.

2. The authors discuss whether the examined mutations influence filament structure. However, the manuscript provides almost no discussion of how the substituted amino acids differ from the wild-type residues in terms of charge, hydrophobicity, steric properties, or other physicochemical parameters. These differences may plausibly impact filament architecture. I strongly encourage the authors to expand the discussion to include these considerations.

As suggested, we have included a new paragraph (lines 463-482) in the revised manuscript to discuss how different mutation types affect filament structure.

Minor comments:

1. Mutation data availability

Sequencing data for all mutations should be deposited in an accessible public database.

The genotyping for all cases in this study was confirmed by Sanger sequencing. The data are now available in Supplementary Fig. 1a and the Source Data file.

2. Extended Data Fig. 1

The caption for panel "c" should be expanded and clarified if this result is retained in the final version of the manuscript.

As suggested above, this panel has been removed and is no longer present in the revised manuscript.

3. Lines 548–553:

The authors should specify the secondary antibodies used, including vendor

information and working dilutions.

The related immunoblot and immunohistochemistry data are not present in the revised manuscript.

4. Mass spectrometry data
Although the mass spectrometry (MS) results appear technically sound, the rationale for performing MS exclusively on the F64S variant is unclear. The manuscript does not explain the purpose of the MS experiment nor how the results complement or validate the cryo-EM findings. This requires clarification.

We fully agree with the reviewer that the rationale for performing mass spectrometry exclusively on the F64S variant was not sufficiently explained in the original manuscript. MS analysis was not performed for the other cases due to limited tissue availability. Filaments from the ATTRv-F64S case were obtained from vitreous humor samples during a vitrectomy, which provided sufficient material for MS analysis. In contrast, filaments from the other cases were extracted from gastrocnemius muscle biopsies, which were residual specimens from routine pathological examinations and were not enough for MS after cryo-EM analysis. We have now clarified this in the Results section (lines 368–369) of the revised manuscript.

Inspired by the recently published study on skin-derived ATTRv-F64S filaments (PMID: 41402329), we further analyzed our mass spectrometry data. In addition to confirming the presence of both wild-type TTR and the TTR-F64S mutant in the deposits, we performed a quantitative analysis of their relative abundances by comparing the peak areas of peptides spanning the mutation site. The results showed that the mutant TTR was predominant, which supports our cryo-EM map–based prediction (lines 370-377, Supplementary Fig. 1d, 1e).

In general, the manuscript can be accepted for publication after corrections to the comments.

Reviewer #1 (Remarks to the Author):

The authors could address several of the issues raised in the last evaluations. However, three problems remain and are not dealt with in an appropriate fashion.

We thank the reviewers for their constructive and helpful comments, which we feel have contributed to an improved manuscript.

1. The use of the term filament is still inappropriate.
In their rebuttal letter, the authors claim to have substituted the term "filament" with "structure" wherever appropriate, but I cannot see that this was done - except in a few cases. The manuscript is still full of the term "filament", and this is problematic for two reasons: First, the authors seem to uniformly write "amyloid filament" when meaning "amyloid fibril". This gives the impression that they do not mean "amyloid fibril" but something else, perhaps a substructure of an amyloid fibril, such as a protofilament. For clarity, the phrase "amyloid fibril" should be used wherever appropriate; that is, in most cases. Second, the term "filament" implies a linear single object. Following my request for additional analysis (new Fig. 7), the authors now admit that many of their reconstructions do not represent discrete filaments but different structures within the same filament. In these cases, reconstructions need to be referred to as "reconstructions" or "structures" but not as "filaments".

Thank you for this important comment. In our previous revision, we did not fully understand the reviewer's concern and primarily focused on substituting "filament" with "structure" in contexts referring to reconstructions. In the current revision, we have replaced all "filament" with "fibril," as recommended by the reviewer. The term "filament" now only appears within the fixed term "protofilament".

2. The assignment of structures in SI Fig. 8 or the revised manuscript.
The assignment of structures could neither be followed in the previous version nor in the revised manuscript. I now explicitly require a separate methods section describing the assignment. This section should state the age cut off values for assignment to early and late onset and the parameters and cut off values used for the assignment into "wildtype dominated", "mixed" or "mutant dominated". In addition, I request a table listing for each mutant and reconstruction the age of onset, the assignment towards early and late onset, and, for the assignment into "wildtype dominated", "mixed" or "mutant dominated", the relevant parameter values per reconstruction, and the resulting assignment into "wildtype dominated", "mixed" or "mutant dominated".

Thank you for this important suggestion. The assignment of structures has now been clarified in the revised manuscript.

A separate Methods section entitled "*Deep-learning-based protein variants assessment*" has been added. In this section, we now state a threshold of 50 years to distinguish early- from late-onset cases, in accordance with established consensus (PMID: 23483184, 31907599). We also have clarified the criteria for classifying structures as wild-type–dominant, mutant–dominant, or mixed:

- Only structures in which ≥70% of the central 30 rungs were predicted as either wild-type or mutant amino acids in both CA and MA were assigned to the corresponding dominant category, and all others were classified as mixed. The 70% threshold was derived from analyses of wild-type control maps, in which the wild-type amino acid frequencies exceeded 70% at the vast majority of sites analyzed in this study.

- As local resolution varies in cryo-EM and affects prediction accuracy, only sites with wild-type frequencies >70% in control maps from both CA and MA are included in subsequent analyses. Based on this criterion, ATTRv-K35N is excluded (MA < 70%), likely due to its location at the edge of the fibril core and its long side chain, which reduce prediction reliability.

- Because the predictions cannot distinguish certain similar amino acids, in some cases, amino acids with similar side chains to the target residue were grouped together during the calculation of prediction frequencies. Details are provided in Supplementary Table 8.

As suggested, Supplementary Table 8 has been added. This table lists, for each mutant and reconstruction, the age of onset, the assignment to early-onset or late-onset, and the parameters used for classification into wild-type–dominant, mutant–dominant, or mixed, together with the resulting assignment.

3. The informational content of Fig. 2b
The colour coding has changed but the blue structures in the two close ups are different from one another and they are also different from the overview image. Therefore, the informational content of this image is still unclear.

Thank you for pointing this out.

The blue structures looked different in the two close-up views because they

were locally realigned in each view, whereas in the overview image they are globally aligned. This local realignment is intended to highlight structural details that are not apparent in the global alignment and was already described in the original figure legend. We have now further clarified this in the revised figure legend.

In addition, we have reorganized the close-up views to improve clarity. One close-up has been moved to Fig. 2a, where residue 83 in both ATTRwt and ATTRv-G83R structures is aligned to highlight the incompatibility of the G83R mutant with the ATTRwt structure. For the remaining close-up in Fig. 2b, the structures are locally realigned based on residues 70-77 to show their local similarity.

Reviewer #2 (Remarks to the Author):

The authors responded to all my questions and comments. In my opinion, the manuscript is suitable for publication.

We thank the reviewer for the positive assessment and for the constructive comments provided throughout the review process.

Reviewer #1 (Remarks to the Author):

The authors have adequately dealt with my comments in most cases. In particular, they now explain how they subdivided the reconstructions into different categories. They also removed the word filament in most cases as required, although they simply substituted it with fibril in almost all of the cases.

We thank the reviewer for the constructive and helpful comments, which we feel have contributed to an improved manuscript.

However, I asked to use the term "structure" instead of filament if the possibility cannot be excluded that two reconstructions show a different regions/segment of the same fibril. This is the case for several reconstructions where the authors now show in their new SI Fig. 7 that the reconstructions represent different segments of the same fibril but not necessarily different fibrils.

Thank you for this important comment. We have carefully re-examined the manuscript and revised the terminology accordingly. We believe that we have addressed all inappropriate uses of "fibril", but we would be grateful if you could let us know should any instances remain.
